# Characterisation of NPFF-expressing neurons in the superficial dorsal horn of the mouse spinal cord

**DOI:** 10.1038/s41598-023-32720-3

**Published:** 2023-04-11

**Authors:** Raphaëlle Quillet, Allen C. Dickie, Erika Polgár, Maria Gutierrez-Mecinas, Andrew M. Bell, Luca Goffin, Masahiko Watanabe, Andrew J. Todd

**Affiliations:** 1 School of Psychology and Neuroscience, Sir James Black Building, University of Glasgow, Glasgow, G12 8QQ UK; 2 Department of Anatomy, Hokkaido University School of Medicine, Sapporo, 060-8638 Japan

**Keywords:** Cellular neuroscience, Somatosensory system

## Abstract

Excitatory interneurons in the superficial dorsal horn (SDH) are heterogeneous, and include a class known as vertical cells, which convey information to lamina I projection neurons. We recently used pro-NPFF antibody to reveal a discrete population of excitatory interneurons that express neuropeptide FF (NPFF). Here, we generated a new mouse line (NPFF^Cre^) in which Cre is knocked into the *Npff* locus, and used Cre-dependent viruses and reporter mice to characterise NPFF cell properties. Both viral and reporter strategies labelled many cells in the SDH, and captured most pro-NPFF-immunoreactive neurons (75–80%). However, the majority of labelled cells lacked pro-NPFF, and we found considerable overlap with a population of neurons that express the gastrin-releasing peptide receptor (GRPR). Morphological reconstruction revealed that most pro-NPFF-containing neurons were vertical cells, but these differed from GRPR neurons (which are also vertical cells) in having a far higher dendritic spine density. Electrophysiological recording showed that NPFF cells also differed from GRPR cells in having a higher frequency of miniature EPSCs, being more electrically excitable and responding to a NPY Y1 receptor agonist. Together, these findings indicate that there are at least two distinct classes of vertical cells, which may have differing roles in somatosensory processing.

## Introduction

The spinal dorsal horn contains the first synapse for pathways that underlie perception of pain, skin temperature and itch^1,2^. Primary afferent input to this region is highly ordered, with nociceptive, thermoreceptive and pruritoceptive afferents terminating in specific zones within the superficial dorsal horn (SDH; laminae I-II). The main ascending pathway that conveys this information to the brain is the anterolateral system (ALS). ALS projection neurons are concentrated in lamina I and the lateral spinal nucleus, and present at a lower density in deeper laminae (III-VIII), the lateral white matter and around the central canal. However, despite the presence of ALS cells in lamina I, the vast majority (~ 99%) of SDH neurons have axons that remain within the spinal cord, and are classified as interneurons^3–6^. Around 75% of these are excitatory glutamatergic cells^7^.

Findings from anatomical, electrophysiological and transcriptomic studies have revealed that SDH excitatory interneurons are heterogeneous^2,8–11^. For example, Grudt and Perl^9^ combined whole-cell patch-clamp recording with morphological reconstruction and described 3 classes in lamina II (vertical, radial and transient central cells) that were subsequently shown to be excitatory^12,13^. We have recently identified 7 largely non-overlapping neurochemical populations among SDH excitatory interneurons^13–18^. Five of these are defined by expression of a neuropeptide (neurotensin, neurokinin B, cholecystokinin, substance P or neuropeptide FF), and one by the presence of gastrin-releasing peptide receptor mRNA (for convenience we refer to these as GRPR cells). The seventh group consists of neurons that express green fluorescent protein (GFP) under control of gastrin-releasing peptide (GRP) in a BAC transgenic mouse (GRP::GFP)^19^. Three of these classes (those expressing neurotensin, cholecystokinin and neurokinin B) are found in lamina IIi-III, and overlap with cells that possess protein kinase Cγ (PKCγ). We have recently reported a correlation between neurochemistry and morphology for 3 of the other 4 classes, since GRP-GFP, substance P and GRPR cells largely correspond to central cells, radial cells and vertical cells, respectively^13,20^.

The NPFF cells are located in laminae I and II and correspond to the Glut9 cluster identified by Häring et al^8^. Relatively little is known about the role of NPFF, although it is thought to exert an anti-nociceptive action through NPFF-2 receptors expressed on primary afferent and dorsal horn neurons^21,22^. We identified the NPFF cells with an antibody against the precursor protein pro-NPFF, and estimated that they account for ~ 6% of the excitatory neurons in the SDH^14^. However, until now, little was apparently known about these cells, and we have therefore generated a mouse line in which Cre recombinase is knocked into the *Npff* locus, allowing them to be targeted. The aim of this study was to characterise the NPFF cells in the SDH by using this mouse line and to test the prediction that these would constitute a morphologically and physiologically distinct population.

## Results

### Distribution of tdTomato-positive cells in the NPFF^Cre^;Ai9 mouse

**Figure 1:**
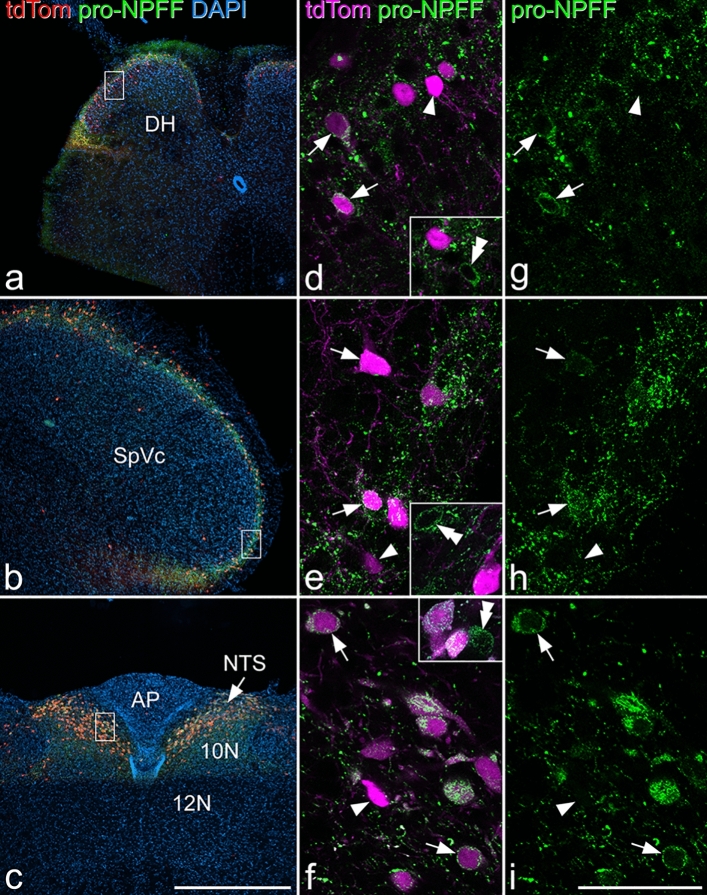
tdTomato-positive cells in the NPFF^Cre^;Ai9 mouse. (**a**), (**b**) and (**c**) show low-magnification views through the spinal cord, spinal trigeminal nucleus and nucleus tractus solitarius (NTS), respectively. Cells labelled with tdTomato (tdTom; red) are present at a high density in the superficial parts of the spinal and trigeminal dorsal horns and in the NTS. (**d**-**i**) higher magnification images from regions shown in the boxes in (**a**–**c)**. TdTomato is shown in magenta, and pro-NPFF immunostaining in green. Several td-Tomato-containing cells are pro-NPFF-immunoreactive, and some of these are marked with arrows. Arrowheads in these images indicate tdTomato-containing cells that do not contain detectable pro-NPFF. Insets in (**d**–**f)** include cells that are pro-NPFF-immunoreactive, but do not contain tdTomato (double arrowheads). Quantitative analysis of the superficial dorsal horn of the spinal cord revealed that 38% of tdTomato-labelled cells were pro-NPFF-immunoreactive, while 83% of pro-NPFF-immunoreactive cells were tdTomato-positive. Images in (**a**), (**b**) and (**c**) are maximum intensity projections of 4, 7 and 14 optical sections, respectively, each at 4 μm z-separation. Main images and insets in (**d**), (**g**), (**e**) and (**h**) are projections of 2 images at 1 μm z-separation. Main images in (**f**) and (**i**) are single optical sections, while insets are projections of 3 optical sections at 1 μm z-separation. Scale bars: **a**-**c** = 500 μm, **d**-**i** = 50 μm. Abbreviations: AP, area postrema; DH, dorsal horn; NTS, nucleus tractus solitarius; SpVc, spinal trigeminal nucleus caudal part; 10N, motor nucleus of vagus; 12N, hypoglossal nucleus.

NPFF-expressing cells have a restricted distribution in rodent CNS, being found in the nucleus of the solitary tract (nucleus tractus solitarius, NTS), and in the SDH of the spinal cord and spinal trigeminal nucleus^14,23–26^. We therefore examined sections from brain and spinal cord of NPFF^Cre^ mice crossed with the Ai9 reporter line, in which Cre-dependent excision of a STOP cassette results in expression of tdTomato. This should result in tdTomato labelling of cells that express Cre at any time during development. As expected, we found a high density of tdTomato-positive cells in laminae I–II of the spinal cord (Fig. 1a), the corresponding region in the spinal trigeminal nucleus (Fig. 1b), and in the NTS (Fig. 1c). Immunohistochemistry revealed that many of the tdTomato-positive cells in these areas were pro-NPFF-immunoreactive^14^ (Fig. 1d–i). TdTomato-labelled axons were present in the lateral parabrachial area (LPb; Fig. S1a,b), and these are likely to originate from cells in NTS, many of which project to LPb^27^. Unexpectedly, tdTomato-positive cells were seen at low density (less than 50 cells per mm^2^ in 100 μm thick sections) in areas not thought to contain NPFF-expressing cells, including caudatoputamen, superior colliculus, neocortex, hippocampus and cerebellar cortex (Fig. S1c–g). Cells in these areas were invariably negative for pro-NPFF-immunoreactivity*.*

Although many of the tdTomato cells in the NTS, spinal trigeminal nucleus caudalis and spinal dorsal horn contained pro-NPFF-immunoreactivity, within each of these regions some tdTomato cells were not pro-NPFF immunoreactive, while there were also cells that were pro-NPFF immunoreactive and lacked tdTomato (Fig. 1d-i). We quantified labelling in the SDH of the L4 segment of 2 mice using a stereological method^28^ and found that 9.5% (9.2–9.9%) of all neurons were tdTomato-positive. We determined the proportion of tdTomato cells that were pro-NPFF-immunoreactive, and vice versa, in these animals by examining a mean of 224 tdTomato cells (214–234). We found that 38% (34–42%) of these contained detectable pro-NPFF immunoreactivity while 83% (77–89%) of pro-NPFF-immunoreactive cells were tdTomato-positive. These values are consistent with our previous finding that 4.7% of all SDH neurons are pro-NPFF-immunoreactive^14^. We also immunostained for Pax2, which is expressed by all dorsal horn inhibitory interneurons^29,30^ and found that none of the tdTomato cells were Pax2-immunoreactive (Fig. S2). Since we have shown that excitatory cells account for 74.2% of neurons in laminae I-II^7^, the tdTomato population corresponds to 12.8% of excitatory neurons in this region.

### Labelling of cells following intraspinal injection of AAVs

The finding that only ~ 40% of tdTomato-positive cells in the SDH were pro-NPFF-immunoreactive could result from failure of the pro-NPFF antibody to detect some cells with genuine NPFF expression or from capture of cells that do not express NPFF in adult animals. The latter option could result from either transient NPFF expression which was captured by the reporter line^15,31^ or from "ectopic" expression of Cre by cells that never expressed NPFF. Conversely, the lack of tdTomato in some pro-NPFF-immunoreactive cells is likely to have resulted from failure of these cells to express sufficient Cre to allow excision of the STOP cassette. We used two approaches to explore these possibilities: (1) intraspinal injection of AAV coding for Cre-dependent GFP (AAV.flex.GFP), which avoids the problem of transient expression^15,31^, and (2) fluorescence in situ hybridisation (FISH), which should reveal the relation of *iCre* and *Npff* mRNAs.

**Figure 2:**
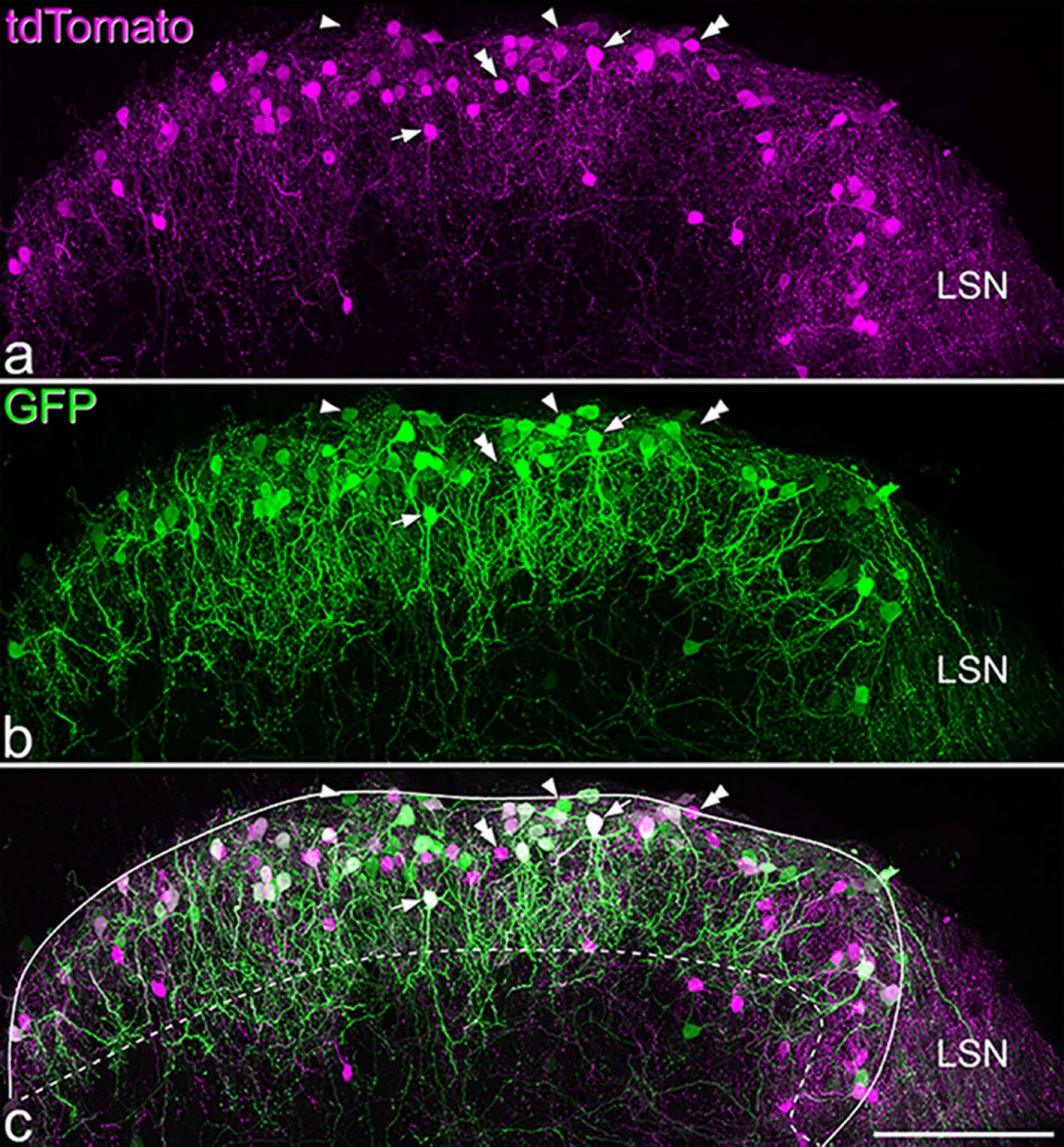
The distribution of tdTomato- and GFP-labelled cells in the L3 segment of the spinal cord of a NPFF^Cre^;Ai9 mouse that had received intraspinal injections of AAV.flex.GFP. **a** and **b** show that both tdTomato- and GFP-positive cells are largely restricted to the superficial dorsal horn. **c**: the merged image reveals that many cells contain both fluorescent proteins (some indicated with arrows), while some cells are positive for only GFP or tdTomato (some of these are marked with single or double arrowheads, respectively). Dendritic labelling is more prominent in the GFP image, and dendrites of these cells commonly project ventrally into the deeper regions of the dorsal horn. Some labelling with each fluorescent protein is seen in the lateral spinal nucleus (LSN), but labelled cell bodies are seldom seen here. We found that 48% of labelled cells contained both fluorescent proteins, 30% only contained GFP and 22% contained only tdTomato. The solid and dashed lines in **c** indicate the edge of the grey matter and the approximate border between laminae II and III, respectively. Images are projections of 58 optical sections at 1 μm z-separation. Scale bar = 100 μm.

**Figure 3:**
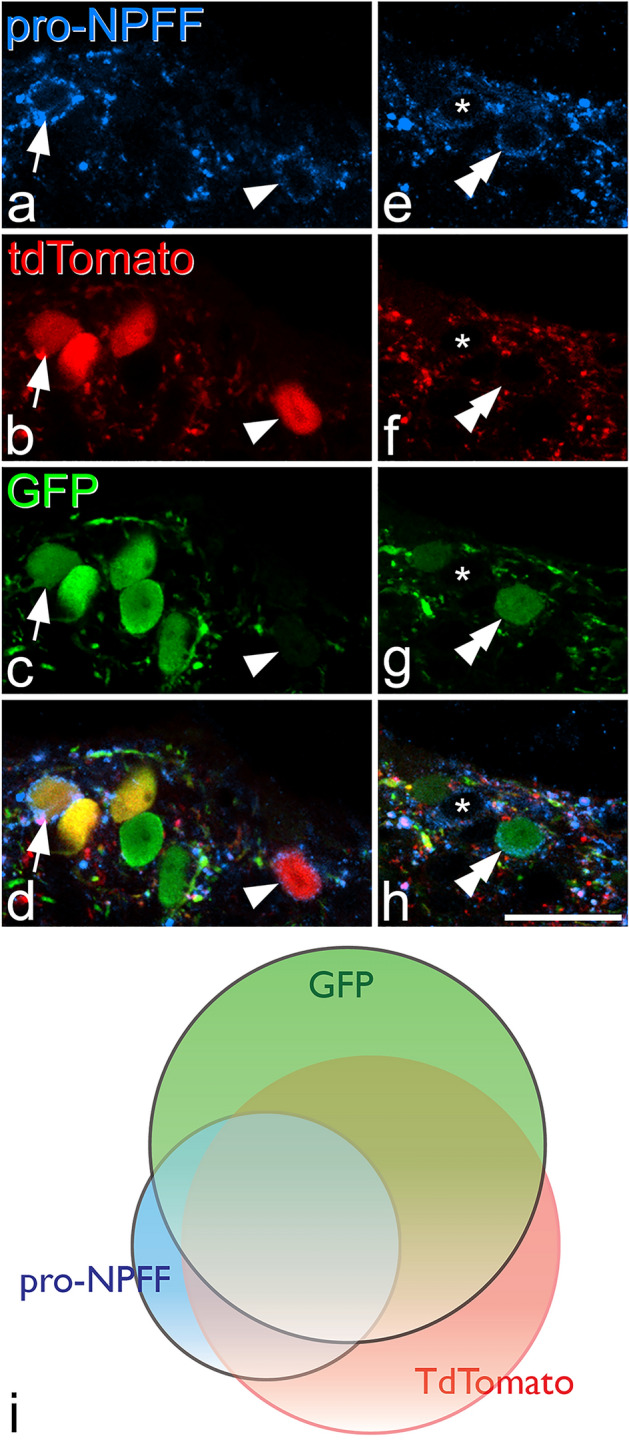
The relation of pro-NPFF immunoreactivity to expression of tdTomato and GFP in a NPFF^Cre^ mouse following intraspinal injection of AAV.flex.GFP. (**a**)–(**d**): show part of the section illustrated in Fig. 2 scanned to reveal immunostaining for pro-NPFF (blue) as well as expression of tdTomato (red) and GFP (green). Two pro-NPFF-positive cells can be identified by the presence of immunoreactivity in the perikaryal cytoplasm, which surrounds the unstained nucleus. One of these cells (arrow) contains both fluorescent proteins, while the other (arrowhead) contains tdTomato but lacks GFP. (**e**)–(**h**): a nearby field from the same section. Again, this contains two pro-NPFF-positive cells, but in this case one is GFP + /tdTomato-negative (double arrowhead), and the other (asterisk) lacks both fluorescent proteins. Among pro-NPFF-immunoreactive cells, 67% were labelled with both GFP and tdTomato, 7.7% only with GFP, 13.5% only with tdTomato, while 11.9% did not contain either fluorescent protein. All images are projections of 2 optical sections at 1 μm z-separation. (**i**): Venn diagram showing the extent of overlap of cells labelled with GFP (green), tdTomato (pink) or pro-NPFF-immunoreactivity (blue). Scale bar for (**a**)–(**h**) = 10 μm.

Following intraspinal injection of AAV.flex.GFP into NPFF^Cre^;Ai9 mice, GFP-labelled cells were largely restricted to the SDH (Fig. 2). However, we found that although there was considerable co-localisation of GFP and tdTomato, many cells were labelled with only one fluorescent protein. Among all fluorescent protein-labelled cells identified in 4 mice (mean 233, range 187–294), 48% (46–51%) contained both fluorescent proteins, 30% (25–37%) were only positive for GFP, while 22% (17–24%) only contained tdTomato. When we examined pro-NPFF immunoreactivity in this tissue, we found that 67% (63–70%) of pro-NPFF-positive cells were labelled with both GFP and tdTomato, 7.7% (5.4–14.5%) only with GFP, 13.5% (12–14.9%) only with tdTomato and 11.9% (9.5–14.3%) were not labelled with either fluorescent protein (Fig. 3). Surprisingly, we found that only 42% (32–45%) of cells with tdTomato and 35% (26–39%) of those with GFP contained detectable pro-NPFF (Fig. 3). None of the GFP-labelled cells and only a single tdTomato-labelled cell (out of 368 cells examined) were Pax2-immunoreactive (Fig. S2). Neither reporter nor viral strategies were therefore able to capture all of the NPFF-expressing cells, although both tdTomato- and GFP-labelled cells in the NPFF^Cre^;Ai9 mice injected with AAV.flex.GFP were restricted to the SDH, where NPFF cells are known to occur^8,14,32–34^. This suggests that while functional levels of Cre in this line may be largely restricted to NPFF-expressing neurons, there is a relatively low level of Cre recombinase in some of these cells, which is insufficient to drive expression with either the reporter or viral strategy.

### Fluorescent in situ hybridisation

**Figure 4:**
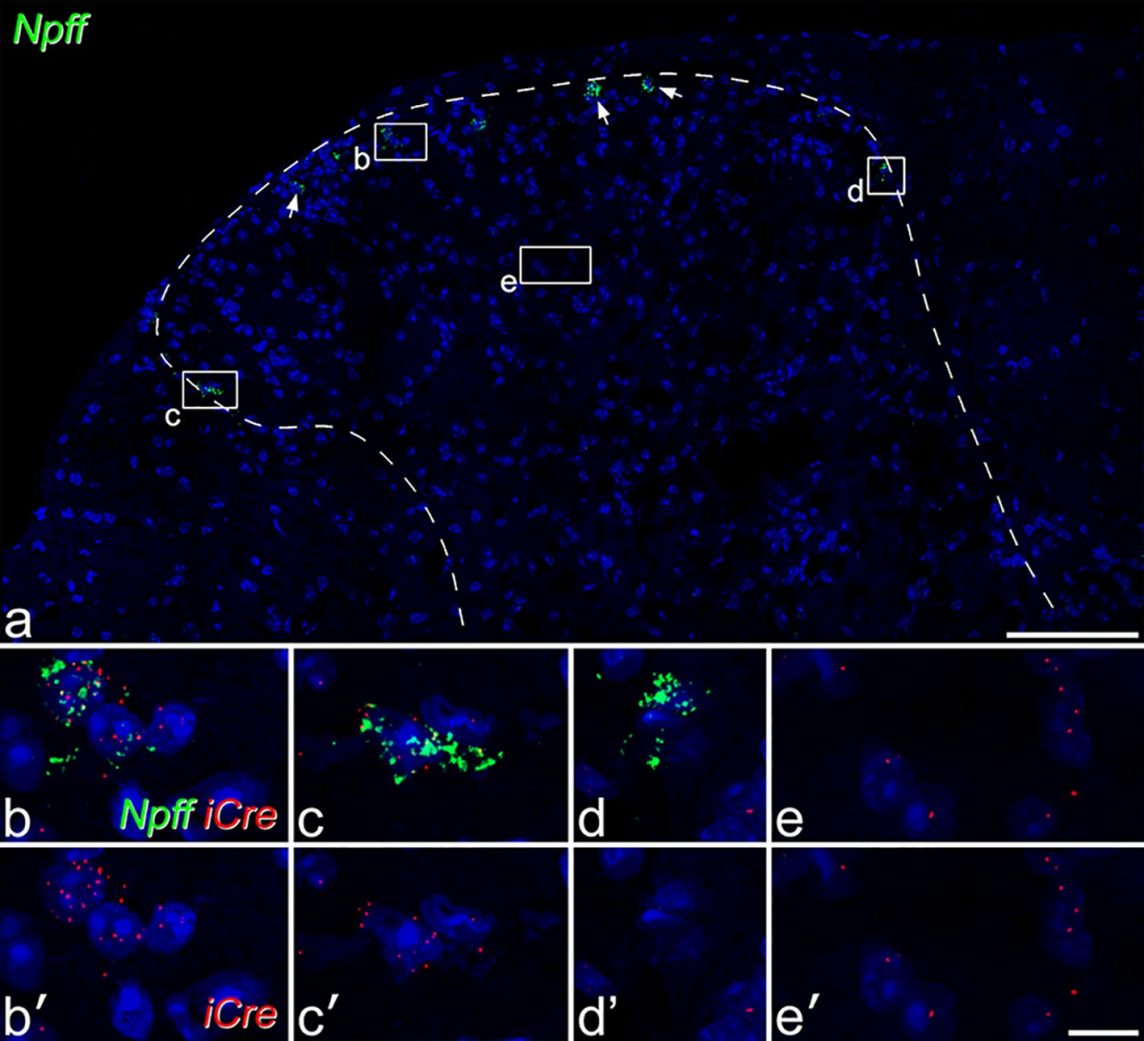
Fluorescence in situ hybridisation labelling for *iCre* and *Npff* in the lumbar spinal cord. (**a**) shows labelling for *Npff* mRNA (green) in a section counterstained with NucBlue (blue). Several *Npff*-positive cells are present in the superficial dorsal horn, and some of these are indicated with arrows. Boxes indicate the locations of the regions shown in (**b**)–(**e**). Note that labelling for *iCre* mRNA is not visible at this magnification. (**b**)–(**e**): higher magnification views showing the relationship between mRNAs for *Npff* (green) and *iCre* (red). Most of the cells with *Npff* also contain several particles representing *iCre*, and examples are shown in (**b**) and (**c**). However, some *Npff*-positive cells are negative for *iCre*, and an example is shown in (**d**). Throughout the dorsal horn, many cells that lack *Npff* contain a small number of particles corresponding to *iCre* as shown in (**e**). All images are maximum intensity projections of 30 confocal optical sections at 0.5 μm z-spacing. Scale bars: (**a**) = 100 μm, (**b**)–(**e**) = 10 μm.

Because of the partial mismatch between NPFF and the fluorescent proteins, we looked for evidence of ectopic expression of Cre in NPFF-negative cells, and for lack of Cre in some NPFF-expressing cells. We compared the distribution of *Npff* and *iCre* mRNAs with fluorescent in situ hybridisation on tissue from 3 NPFF^Cre^ mice. We had previously shown that in a GRPR^CreERT2^ mouse line^35^, *iCre* mRNA was highly expressed in cells with *Grpr* mRNA, and was essentially restricted to these neurons. In tissue from the NPFF^Cre^ mouse, we observed the expected distribution of *Npff*-positive cells (Fig. 4a), which were restricted to the SDH, as we have previously described^14^. However, we found that although many *NPFF*-positive cells contained several transcripts for *iCre* (Fig. 4b,c), low levels of *iCre* were distributed throughout the spinal cord, and were found in many neurons that lacked *Npff* (Fig. 4e). In addition, some cells with *Npff* contained no *iCre* transcripts (Fig. 4d).

### Relation of cells captured in the NPFF^Cre^ mouse to other neurochemical populations of excitatory interneurons

**Figure 5:**
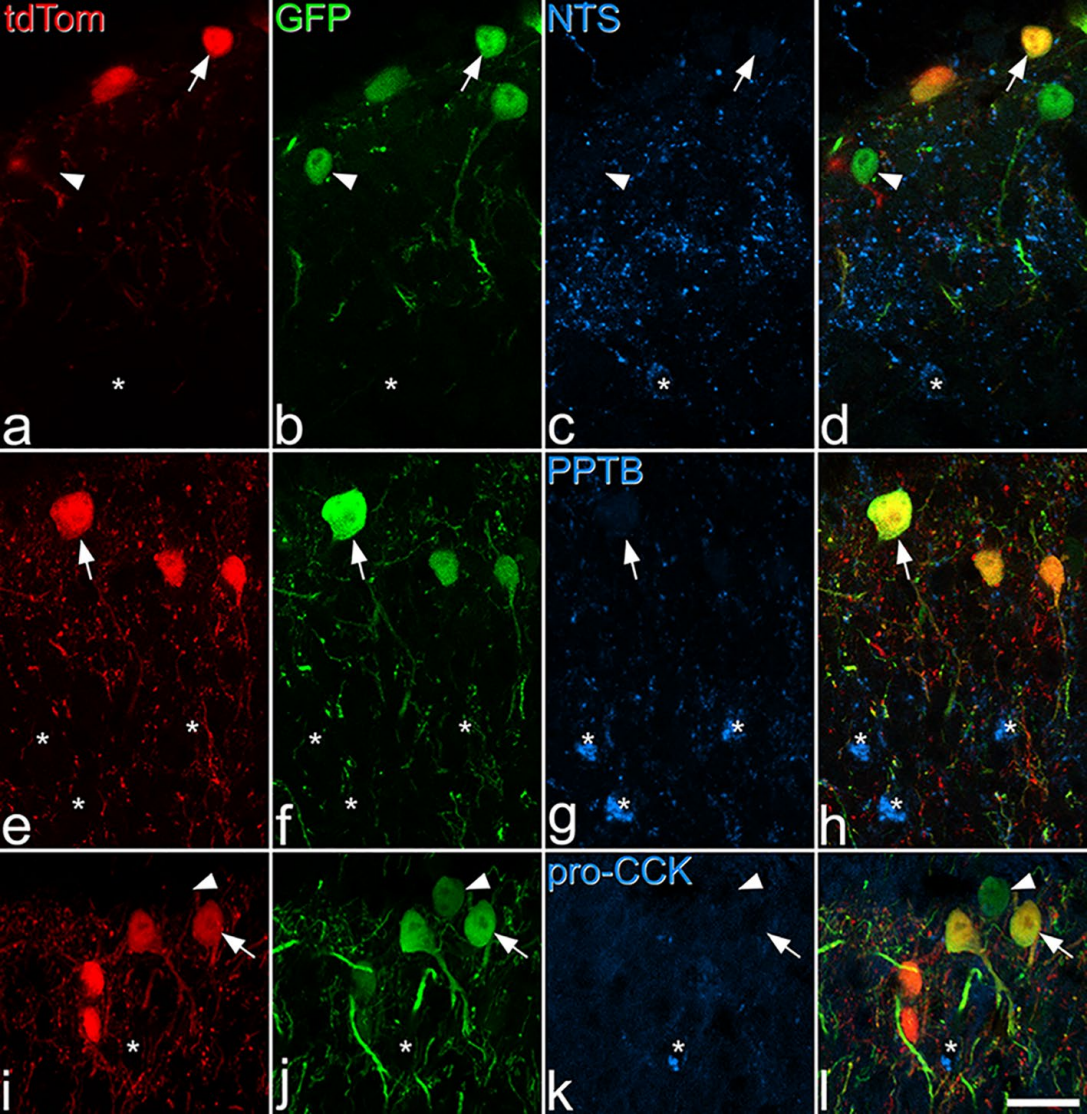
Lack of expression of neuropeptides associated with certain excitatory interneuron populations by NPFF-Cre neurons. Sections from NPFF^Cre^;Ai9 mice that had been injected with AAV.flex.GFP were reacted with antibodies against neurotensin (NTS) (**a**)–(**d**), preprotachykinin B (PPTB) (**e**)–(**h**), or pro-CCK (**i**)–(**l**). Some cells that express both tdTomato and GFP are indicated with arrows, and some that only contain GFP with arrowheads. Cells that are immunoreactive for each of the peptide antibodies are indicated with asterisks. Note that staining for these peptides is located within the perikaryal cytoplasm of labelled neurons, and that there is no overlap with cells containing the fluorescent proteins. A few tdTomato-positive/GFP-negative cells were immunoreactive for neurotensin (1.3%) or pro-CCK (2.3%), whereas none contained PPTB. None of the GFP cells were immunroeactive for any of these (pro)peptides. Images are projections of 3 (**a**–**h**) or 5 (**i**–**l**) optical sections at 1 μm z-spacing. Scale bar = 20 μm.

We immunostained tissue from NPFF^Cre^;Ai9 mice that had received intraspinal injection of AAV.flex.GFP with antibodies against neurotensin, preprotachykinin B (PPTB, the precursor for neurokinin B) or pro-cholecystokinin (the precursor for cholecystokinin, CCK) We found that a very low proportion of cells that were tdTomato-positive/GFP-negative were immunoreactive for neurotensin (mean 1.3%, range 0–2%) or for pro-CCK (mean 2.3%, range 2–3%), while none contained PPTB. None of these peptide markers were seen in any of the cells that were GFP-positive. Examples of immunostaining are shown in Fig. 5.

**Figure 6:**
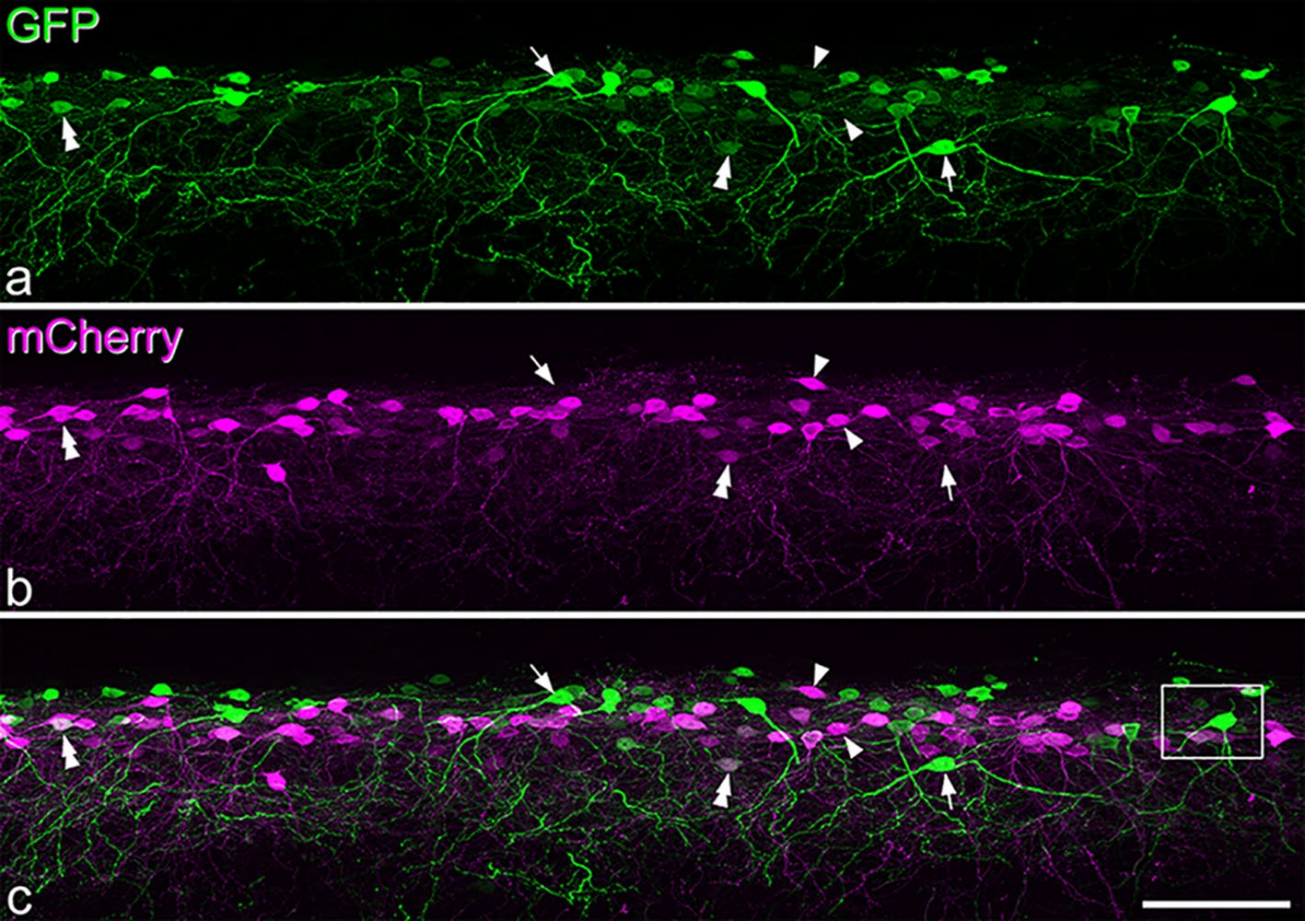
Expression of fluorescent proteins in NPFF^Cre^;GRPR^Flp^ mice that had been injected with AAVs coding for Cre-dependent GFP and Flp-dependent mCherry. (**a**) and (**b**) show immunostaining for GFP and mCherry, respectively, in a sagittal section, and (**c**) is a merged image. There are numerous cells with GFP and/or mCherry expression in the superficial dorsal horn. Arrows, arrowheads and double arrowheads show examples of cells that are labelled only with GFP, only with mCherry or with both GFP and mCherry, respectively. Quantitative analysis showed that 39% of GFP-positive cells also contained mCherry, while 36% of mCherry cells were also GFP-positive. The box shows the approximate position of the field illustrated at higher magnification in Fig. 7. Scale bar = 100 μm.

**Figure 7:**
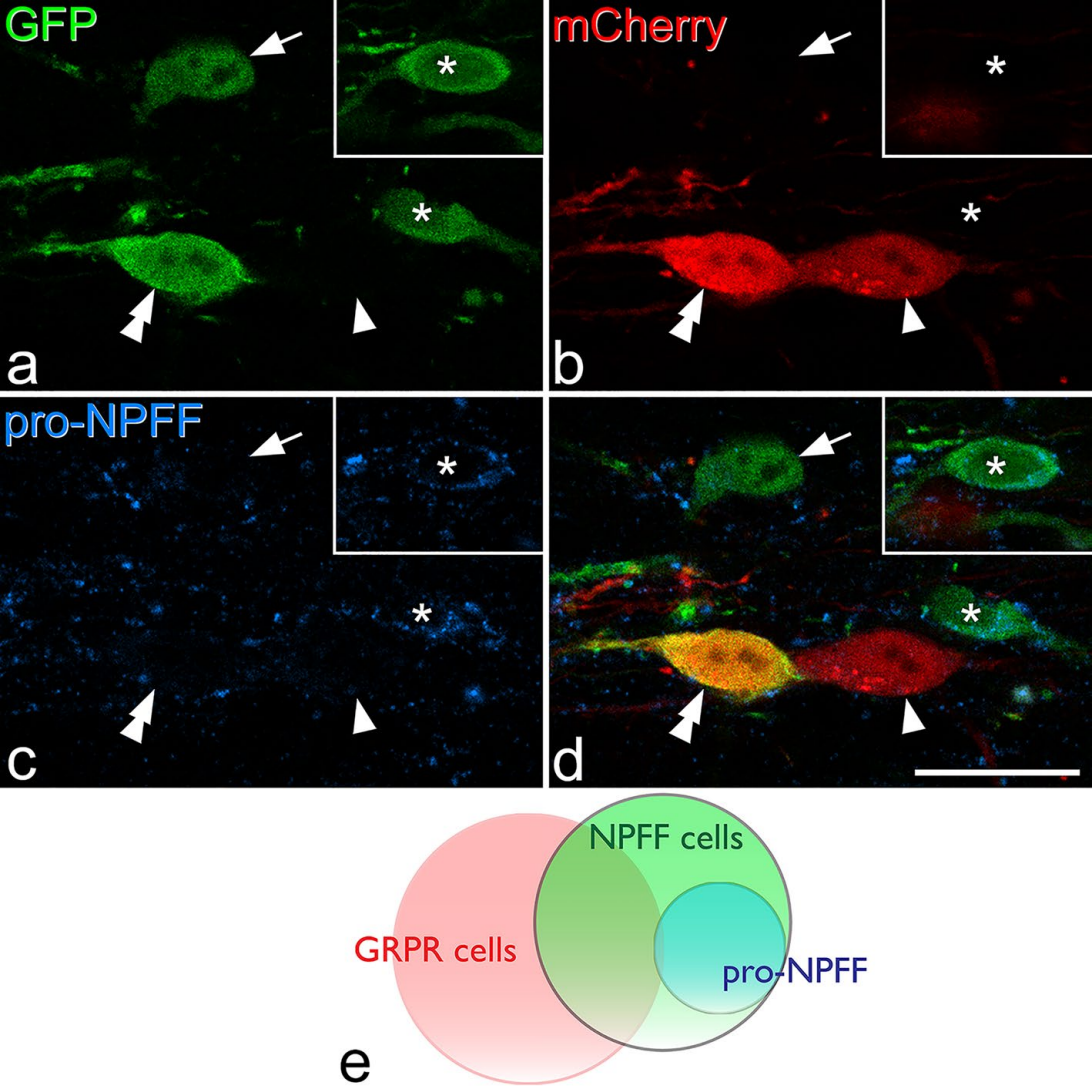
Immunostaining for pro-NPFF in a section obtained from a NPFF^Cre^;GRPR^Flp^ mouse that had been injected with AAVs coding for Cre-dependent GFP and Flp-dependent mCherry. (**a**–**c**) show staining for GFP (green), mCherry (red) and pro-NPFF (blue) in a single confocal optical section, while (**d**) shows a merged image. The arrow and asterisk mark cells that are positive for GFP and negative for mCherry. The cell indicated with the asterisk contains pro-NPFF, and this is seen more clearly in a different optical section (inset). The cell marked with the arrow lacks pro-NPFF. The cells indicated with single and double arrowheads are both positive for mCherry. One of them (double arrowheads) is also labelled with GFP, while the other (single arrowhead) is not. Both of these cells lack pro-NPFF-immunoreactivity. (**e**) Venn diagram showing the relationship between cells labelled with each fluorescent protein and pro-NPFF immunoreactivity. pro-NPFF was present in 0.4% of GRPR cells (labelled with mCherry), 25% of NPFF cells (labelled with GFP) and 42% of the cells that were GFP-positive/mCherry-negative. Scale bar = 20 μm.

GRPR-expressing excitatory interneurons^18^ cannot be revealed with immunohistochemistry, due to lack of reliable antibodies against GRPR. To look for overlap between GRPR cells and those captured by the NPFF^Cre^ mouse, we crossed this line with a GRPR^Flp^ mouse^18^ and injected AAVs coding for Cre-dependent GFP and Flp-dependent mCherry into the spinal cord. The distribution of mCherry-positive cells was very similar to that seen when AAVs coding for Cre-dependent constructs were injected into GRPR^CreERT2^ mice^18^, and cells labelled with each fluorescent protein were largely restricted to the SDH (Fig. 6). We analysed a mean of 383 fluorescent-labelled cells (355–409, n = 4 mice) and found that 39% (28–44%) of GFP-positive cells were also labelled with mCherry, while 36% (31–44%) of mCherry cells contained GFP (Fig. 6). When we examined pro-NPFF-immunoreactivity, we found that 0.4% (0–0.8%) of mCherry cells were pro-NPFF positive (consistent with our finding that < 1% of GRPR-expressing cells in the SDH contain pro-NPFF^18^). Surprisingly, we found only 25% (20–28%) of GFP-labelled cells were pro-NPFF-immunoreactive, compared to the 35% that contained the peptide when AAV.flex.GFP was injected in the NPFF^Cre^;Ai9 cross (Fig. 7). This difference may result from a slightly altered pattern of viral transfection when two different AAVs are injected simultaneously. We found that 42% (29–50%) of cells that were GFP-positive/mCherry-negative in these experiments were pro-NPFF-immunoreactive (Fig. 7). These results show that the NPFF^Cre^ mouse captures a population of excitatory neurons that include those with detectable pro-NPFF protein, as well as some of those belonging to a previously identified population of GRPR-expressing cells, in which pro-NPFF cannot be detected^18^.

### Morphology of NPFF cells

**Figure 8:**
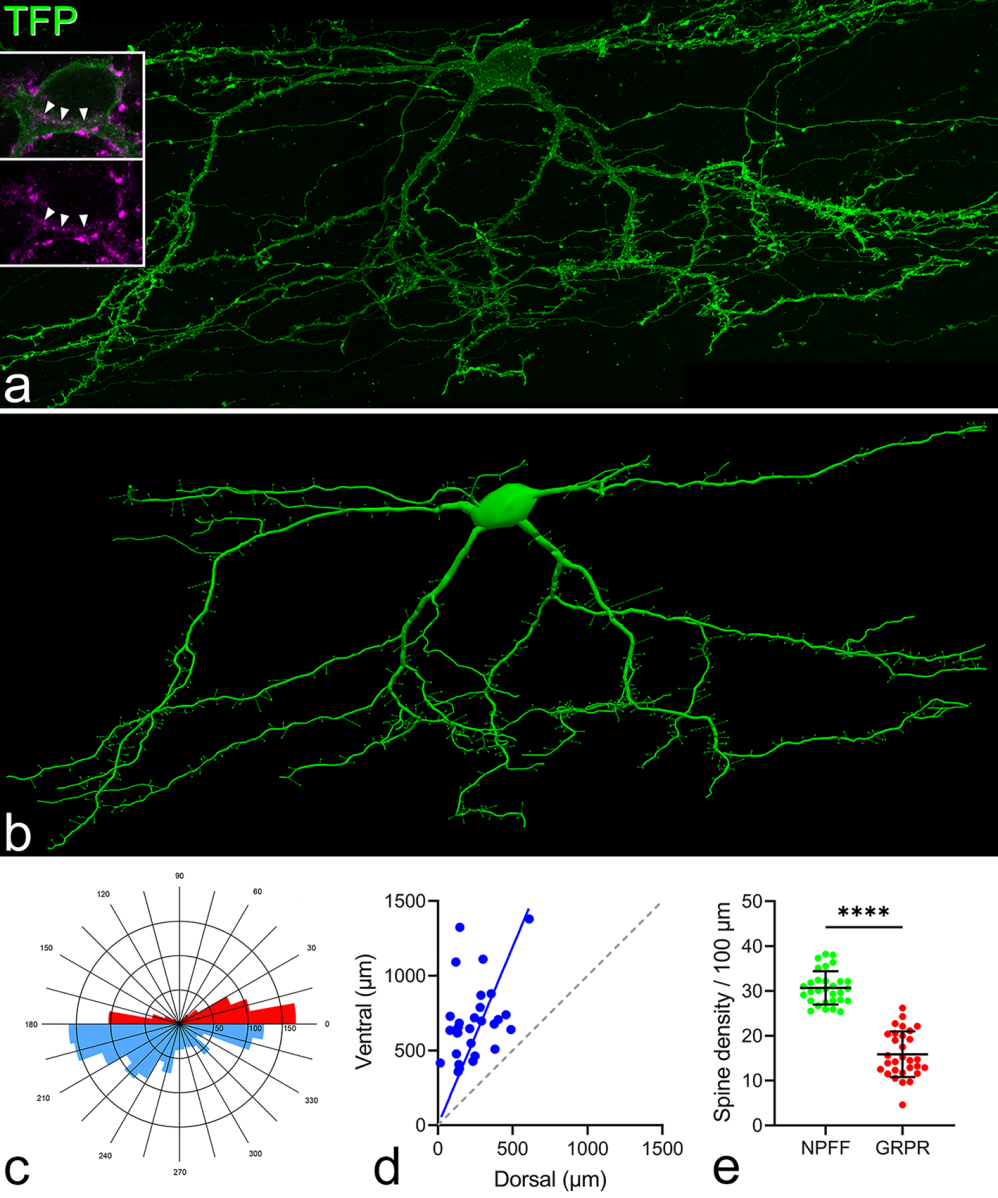
Dendritic morphology of NPFF cells revealed with the viral Brainbow technique. (**a**): a maximum intensity projection (99 optical sections at 0.5 μm z-separation) showing part of a sagittal section from a NPFF^Cre^ mouse that had received intraspinal injections of Brainbow AAVs. The section has been scanned to reveal TFP (green), and examination of confocal images showed that this was the only labelled cell within this field. Insets show labelling for TFP (green) and immunostaining for pro-NPFF (magenta) in a more restricted projection (4 optical sections). The cell body contains pro-NPFF immunoreactivity, which occupies part of the perikaryal cytoplasm, and this is marked by arrowheads. There are also pro-NPFF-immunoreactive profiles outside the cell body, and these are likely to be pro-NPFF-containing axons that may be forming synapses on the Brainbow-labelled cell. (**b**): a Neurolucida reconstruction of the cell shown in (**a**). Positions of dendritic spines are shown, although the sizes of the spine heads and shapes of spine necks in this drawing do not represent the actual sizes and shapes of these structures. Note the high density of dendritic spines on this cell. (**c**): a polar histogram for this cell. Dorsally directed dendrites are shown in red and ventrally directed dendrites in blue. (**d**): A plot of the ratio of the lengths of ventrally-directed (ventral) to the lengths of dorsally-directed (dorsal) dendrites obtained from polar histograms for the 30 NPFF cells analysed in this study. The blue line shows the mean ratio for these cells, while the black dashed line corresponds to a ratio of 1. (**e**): Comparison of the dendritic spine density between the 30 NPFF cells labelled with the Brainbow technique and 30 GRPR cells labelled with the same method from Polgár et al^18^. Scale bar (**a**, **b**) = 50 μm.

Grudt and Perl^9^ identified 3 morphological classes among excitatory interneurons in lamina II: vertical, transient central and radial cells. Vertical cells are commonly located in lamina IIo, and have prominent ventral dendrites. We noted that cells labelled following injection of AAV.flex.GFP in the NPFF^Cre^ mouse often had ventrally directed dendrites (Figs. 2b, 6a), suggesting that they may correspond to vertical cells. To examine dendritic morphology of these cells in more detail, we used the viral Brainbow technique^13,18,36^. We combined this with immunostaining with the pro-NPFF antibody, to allow identification of cells that contained detectable levels of the pro-peptide, and therefore definitely expressed NPFF. We reconstructed the dendritic trees of 30 Brainbow labelled cells that were also pro-NPFF-immunoreactive (Figs. 8a,b, S3), and found that virtually all of these corresponded to vertical cells described in previous studies^9,12,18,37–41^. Polar histograms showed that in all cases the lengths of ventrally-directed dendrites exceeded those of dorsally-directed dendrites (Fig. 8c,d). Some of these cells (14 out of 30) had cell bodies that were less than 20 μm below the dorsal white matter, and were therefore likely to have been in lamina I. However, these did not differ morphologically from those located in lamina II, and we therefore use the term "vertical cells" to describe those in both laminae, as we have done previously for the GRPR cells^18^. We noted that although these cells looked very similar to the GRPR-expressing vertical cells that we had analysed previously^18^, they had a relatively high density of dendritic spines, and we therefore compared this with data for GRPR-expressing neurons. The spine density per 100 μm of dendrite was 30.7 ± 3.7, compared to corresponding values of 15.9 ± 5.1 for the GRPR cells^18^, and this difference was highly significant (*P* < 0.0001; t-test; Fig. 8e).

### Electrophysiological properties

As noted above, intraspinal injection of AAV.flex.GFP in NPFF^Cre^ mice labelled many cells that were also captured in the GRPR^Flp^ line, but very few of these contained pro-NPFF (Fig. 7). We therefore carried out electrophysiological studies on NPFF^Cre^;GRPR^Flp^ mice intraspinally injected with AAV.flex.GFP and AAV.FRT.mCherry, and targeted those cells that were GFP-positive/mCherry-negative for whole-cell patch-clamp recording in spinal cord slices. No sex differences were observed, therefore all data presented are a combination of recordings made in tissue from female and male mice. For convenience, we refer to these GFP-positive/mCherry-negative cells as NPFF cells in the context of our electrophysiological studies.

**Figure 9:**
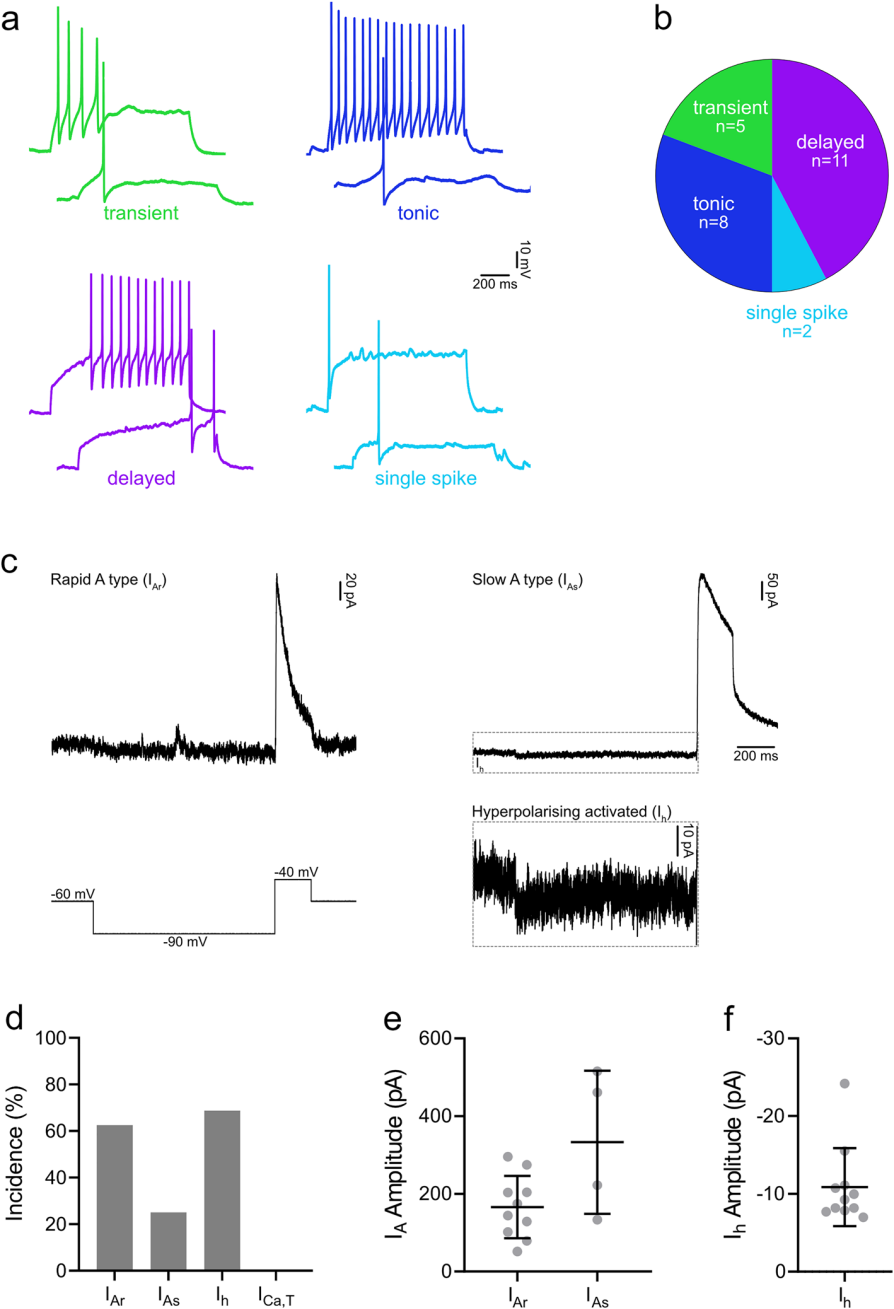
Action potential firing patterns and subthreshold voltage-activated currents in NPFF cells. (**a**) Examples of action potential firing patterns observed in NPFF cells in response to 1 s suprathreshold current injections. (**b**) The most prominent type of firing pattern seen in NPFF cells was delayed firing (11/26; 42.3%), with smaller proportions exhibiting tonic (8/26; 30.8%), transient (5/26: 19.2%) or single spike firing (2/26: 7.7%). (**c**) Representative traces demonstrating the subthreshold voltage-activated currents in NPFF cells that were revealed using a voltage step protocol that hyperpolarised cells from -60 to -90 mV for 1 s and then depolarised cells to -40 mV for 200 ms (bottom left trace). The currents that are revealed by this protocol were classified as rapid (I_Ar_) or slow (I_As_) A-type potassium currents, or hyperpolarising-activated currents (I_h_). The example traces of I_Ar_ (top left) and I_As_ with I_h_ (top right) show an average of 5 traces. The example of I_h_ (dashed outline) is shown at a different y-axis scale (bottom right). (**d**) Almost all NPFF cells displayed I_A_, which was mostly classified as I_Ar_ (10/16; 62.5%), but with some exhibiting I_As_ (4/16; 25.0%). Many cells exhibited I_h_ (11/16; 68.8%), which was typically seen in addition to I_Ar_ (5/16; 31.3%) or I_As_ (4/16; 25.0%). The peak amplitude of I_A_ was 165.7 ± 80.3 and 333.0 ± 184.1 pA for I_Ar_ and I_As_, respectively (**e**) and the amplitude of I_h_, measured during the final 200 ms of the hyperpolarising step, was -10.9 ± 5.0 pA (**f**).

We characterised action potential firing patterns of the NPFF cells in response to depolarising current steps and found that delayed firing was the most prominent type (11/26; 42.3%), with many cells exhibiting tonic (8/26; 30.8%) or transient firing (5/26: 19.2%), but few showing single-spike firing (2/26: 7.7%) (Fig. 9a,b). The membrane properties of these cells are detailed in Table 1, and mean values are described here. Table 1 also includes corresponding data obtained for GRPR cells in our previous study^18^, to allow comparison between these populations. The resting membrane potential of NPFF cells was − 59.0 mV, their input resistance was 749.9 MΩ and their capacitance 10.6 pF. The rheobase current of NPFF cells was 26.9 pA, with the following parameters measured from the first action potential at rheobase: the action potential voltage threshold (defined as the point where the rate of rise exceeded 10 mV/ms) − 35.3 mV, the latency between the onset of the depolarising step to the first action potential was 321.8 ms, base width was 1.4 ms, action potential height (measured as the difference between the voltage threshold and the peak of the action potential) 64.8 mV, and after-hyperpolarisation was − 28.4 mV. Many NPFF cells (14/25; 56.0%) exhibited spontaneous action potential firing (defined as ≥ 1 AP/min), with the mean firing frequency being 0.7 ± 0.8 Hz.

**Table 1:** Comparison between electrophysiological findings for NPFF and GRPR cells.

	NPFF	GRPR	Significance
Capacitance (pF)	10.58 ± 2.2 (n = 31)	9.88 ± 2.7 (n = 256)	0.080
Input resistance (MΩ)	750 ± 307 (n = 31)	767 ± 473 (n = 223)	0.928
Resting membrane potential (mV)	−59.0 ± 8.2 (n = 31)	−58.9 ± 8.9 (n = 218)	0.739
Rheobase (pA)	26.9 ± 20.5 (n = 26)	68.7 ± 43.4 (n = 111)	**< 0.0001**
Action potential threshold (mV)	−35.3 ± 5.4 (n = 26)	-30.0 ± 7.3 (n = 111)	**< 0.0001**
Action potential latency (ms)	321.8 ± 235.8 (n = 26)	349.5 ± 380.0 (n = 111)	0.272
Action potential width (ms)	1.4 ± 0.5 (n = 26)	1.4 ± 0.4 (n = 111)	0.319
Action potential height (mV)	64.8 ± 10.2 (n = 26)	45.2 ± 10.4 (n = 111)	**< 0.0001**
After-hyperpolarisation (mV)	−28.42 ± 5.2 (n = 26)	−25.9 ± 4.9 (n = 111)	**0.024**
I_Ar_ amplitude (pA)	165.7 ± 80.3 (n = 10)	289.7 ± 154.7 (n = 77)	**0.007**
I_As_ amplitude (pA)	333.0 ± 184.1 (n = 4)	92.0 ± 9.4 (n = 3)	0.057
I_h_ amplitude (pA)	−10.9 ± 5.0 (n = 11)	−11.9 ± 7.5 (n = 23)	0.631
sEPSC frequency (Hz)	6.86 ± 6.15 (n = 27)	4.34 ± 5.26 (n = 189)	**0.005**
sEPSC amplitude (pA)	34.3 ± 6.3 (n = 27)	32.8 ± 8.5 (n = 189)	0.063
mEPSC frequency (Hz)	2.35 ± 1.99 (n = 15)	1.02 ± 1.34 (n = 42)	**0.002**
mEPSC amplitude (pA)	30.1 ± 4.1 (n = 15)	27.1 ± 5.4 (n = 42)	**0.023**

Data for GRPR cells was obtained from Polgár et al^18^. Details of the statistical tests are provided in the text. Significant values are in bold.

To investigate subthreshold voltage-activated currents, we used a voltage step protocol that can reveal the presence of 2 types of transient outward current and 2 types of inward current, which are consistent with rapid (I_Ar_) or slow (I_As_) A-type potassium currents (I_A_), and the low-threshold “T-type” calcium current (I_Ca,T_) or the hyperpolarisation-activated (I_h_) current, respectively (Fig. 9c). Almost all cells exhibited I_A_, which was mostly I_Ar_ (10/16; 62.5%), but with some displaying I_As_ (4/16; 25.0%) (Fig. 9d). Many cells were found to have I_h_ (11/16; 68.8%), which was typically seen alongside I_Ar_ (5/16; 31.3%) or I_As_ (4/16; 25.0%). I_Ca,T_ was not detected in any cells. The sizes of these currents are shown in Fig. 9e,f and Table 1.

**Figure 10:**
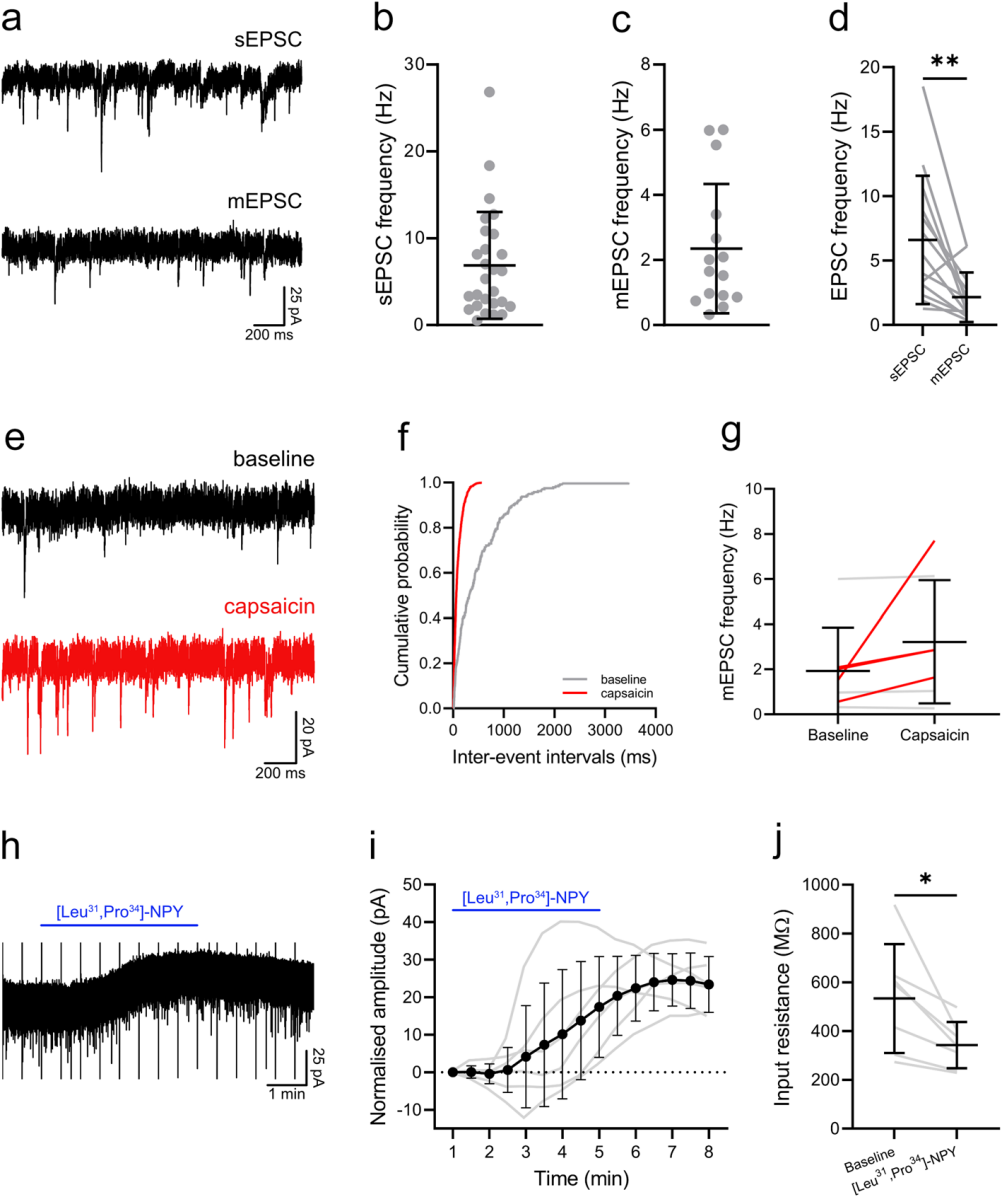
Excitatory synaptic input to NPFF cells and responses to capsaicin and the neuropeptide Y1 receptor agonist, [Leu^32^,Pro^34^]-NPY. (**a**) Example traces of spontaneous (top) and miniature (bottom) EPSCs recorded in the same NPFF cell. The frequency of the sEPSCs was 6.9 ± 6.1 Hz, n = 27 (**b**) and the frequency of the mEPSCs was 2.3 ± 2.0 Hz, n = 15 (**c**). (**d**) In those cells where both sEPSCs and mEPSCs were recorded, the sEPSC frequency was significantly greater 6.6 ± 5.0 vs. 2.2 ± 1.9 Hz; *P* = 0.003, paired t test, n = 13. The functional expression of TRPV1 channels on primary afferent input to NPFF cells was assessed by recording mEPSCs in response to the application of the TRPV1 agonist, capsaicin. (**e**) Representative traces of mEPSCs recorded before (baseline: top) and during application of capsaicin (bottom). (**f**) Example cumulative probability plot demonstrates a significant leftward shift in the distribution of interevent intervals in response to capsaicin (*P* < 0.00001, Kolmogorov–Smirnov 2-sample test). A significant leftward shift in mEPSC interevent intervals, signifying an increase in mEPSC frequency, was seen in 4 of 7 cells treated with capsaicin (**g**). In those cells that responded to capsaicin, the mEPSC frequency was increased from 1.5 ± 0.7 to 3.8 ± 2.7 Hz, n = 4 (red lines), while in the remaining 3 cells (grey lines) mEPSC frequency was 2.4 ± 3.1 and 2.5 ± 3.2 Hz prior to and during capsaicin application, respectively. Responses of NPFF neurons to the neuropeptide Y1 receptor agonist, [Leu^32^,Pro^34^]-NPY, were assessed and an example trace is shown in (**h**). (**i**) All six NPFF cells tested displayed a clear outwards current in response to [Leu^32^,Pro^34^]-NPY, which also resulted in a significant reduction in input resistance from 533.5 ± 223.5 to 342.3 ± 95.0 MΩ, *P* = 0.016, Wilcoxon matched-pairs signed rank test (**j**).

Excitatory synaptic input to NPFF cells was investigated by recording spontaneous (sEPSC) and miniature (mEPSC) excitatory postsynaptic currents, at a holding potential of − 70 mV. The frequency was 6.9 ± 6.1 Hz and 2.3 ± 2.0 Hz, and the amplitude was 34.3 ± 6.3 pA and 30.1 ± 4.1 pA, for sEPSCs and mEPSCs, respectively (Fig. 10a–c, Table 1). In 13 cases we compared sEPSCs and mEPSCs in the same cell and found that the sEPSC frequency was significantly greater than the mEPSC frequency (6.6 ± 5.0 vs. 2.2 ± 1.9 Hz; *P* = 0.003, paired t test, Fig. 10d), suggesting that the NPFF cells received excitatory synaptic input from other cells in the slice that were spontaneously active. The sEPSC amplitude in these cases was also significantly greater than the mEPSC amplitude (35.5 ± 6.3 vs. 30.7 ± 4.2 pA; *P* = 0.005, paired t test).

We investigated whether NPFF cells received input from TRPV1-expressing primary afferents by recording mEPSCs in response to the TRPV1 agonist, capsaicin. Capsaicin caused a leftward shift in the distribution of mEPSC interevent intervals in 4 of 7 cells tested (Fig. 10e, f), which were considered to be capsaicin-sensitive. In these capsaicin-sensitive cells mEPSC frequency was increased from 1.5 ± 0.7 to 3.8 ± 2.7 Hz (Fig. 10g). Expression of the TRPV1 channel in the dorsal horn is restricted to primary afferents, and these results indicate that some NPFF cells receive monosynaptic input from TRPV1-expressing primary afferents.

NPY acting on the neuropeptide Y1 receptor suppresses hypersensitivity in neuropathic and inflammatory pain states^42^, and the Y1 receptor is expressed by cells in the Glut9 cluster^8^. We therefore tested responses of NPFF cells to the Y1 receptor agonist, [Leu^32^,Pro^34^]-NPY, by bath application. All cells tested (6/6) displayed a clear outward current in response to [Leu^32^,Pro^34^]-NPY, the maximum amplitude of which was 27.8 ± 8.8 pA (Fig. 10h,i). [Leu^32^,Pro^34^]-NPY also caused a significant reduction in input resistance, from 533.5 ± 223.5 to 342.3 ± 95.0 MΩ (*P* = 0.016, Wilcoxon matched-pairs signed rank test, Fig. 10j). This indicates that the NPFF cells express functional Y1 receptors.

**Figure 11:**
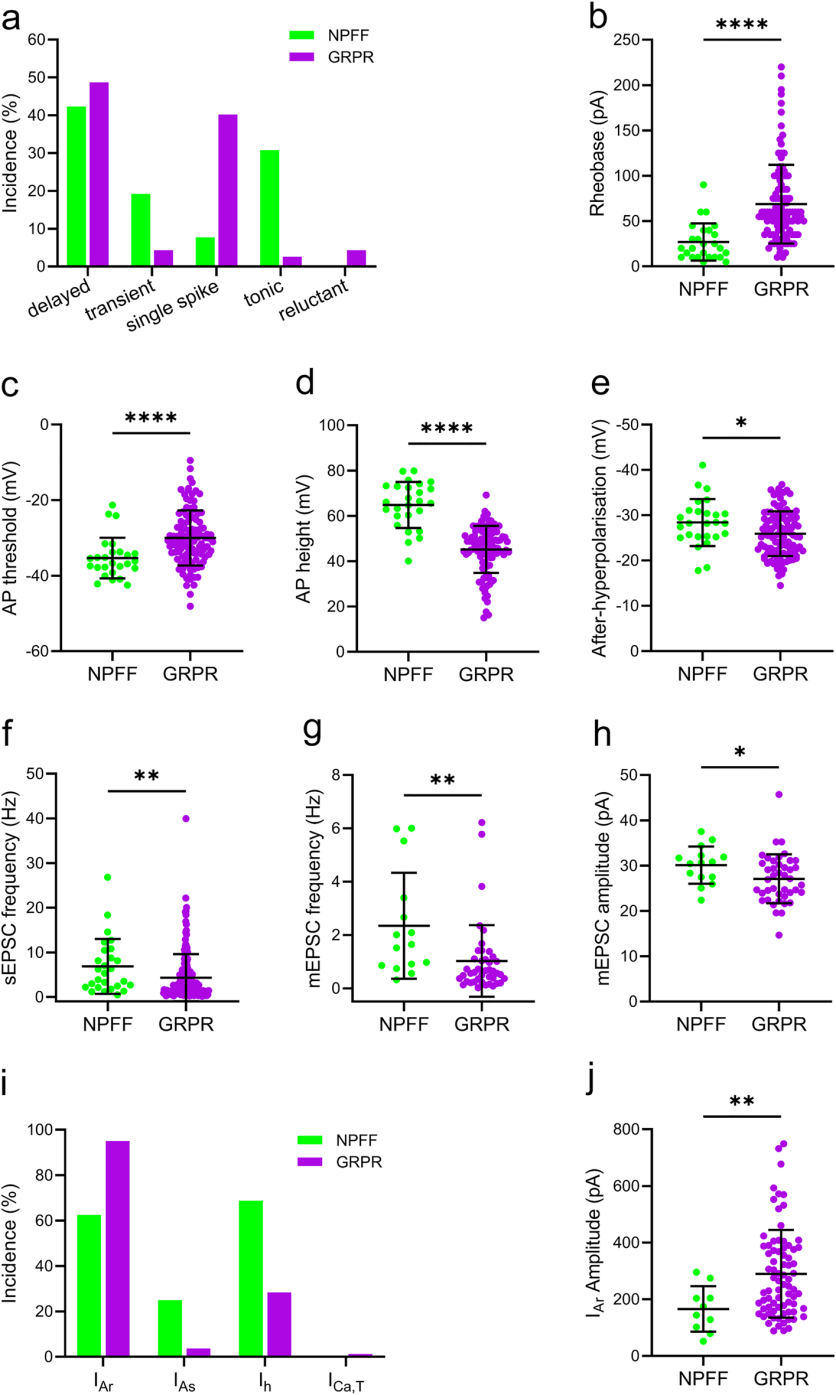
Comparison of the electrophysiological properties of NPFF cells with those of GRPR cells. Data for the GRPR cells was obtained from Polgár et al^18^. (**a**) The incidence of action potential firing patterns differed between NPFF and GRPR cells. NPFF cells exhibited a greater proportion of tonic (30.8 vs. 2.6%) and initial burst firing (19.2 vs. 4.3%) cells and fewer single spike firing cells (7.7 vs. 40.2%). The incidence of delayed firing was similar between both cell types (42.3 vs. 48.7%). NPFF cells were found to have a lower rheobase (26.9 ± 20.5 v.s 68.7 ± 43.4 pA, *P* < 0.0001, **b**), action potential voltage threshold (-35.3 ± 5.4 vs. -30.0 ± 7.3 mV, *P* < 0.0001, **c**) and a greater action potential height (64.8 ± 10.2 vs. 45.2 ± 10.4 mV, *P* < 0.0001, **d**) and after-hyperpolarisation (-28.4 ± 5.2 vs. − 25.9 ± 4.9 mV, *P* = 0.024, **e**). In terms of excitatory synaptic input, sEPSC (6.86 ± 6.15 vs. 4.34 ± 5.26 Hz, *P* = 0.005, **f**) and mEPSC (2.35 ± 1.99 vs. 1.02 ± 1.34 Hz, *P* = 0.002, **g**) frequency in NPFF cells was greater than that in GRPR cells, as was the mEPSC amplitude (30.1 ± 4.1 vs. 27.1 ± 5.4 pA, *P* = 0.023, **h**). (**i**) The incidence of subthreshold voltage-activated currents differed between NPFF and GRPR cells. NPFF cells demonstrated a higher incidence of I_As_ (25.0 vs. 3.7%) and I_h_ (68.8 vs. 28.4%), but a lower incidence of I_Ar_ (62.5 vs. 95.1%). (**j**) The peak amplitude of the I_Ar_ recorded in NPFF cells was significantly lower than for GRPR cells (165.7 ± 80.3 vs. 289.7 ± 154.7, *P* = 0.007). All statistical comparisons made using a Mann Whitney test, except for afterhyperpolarisation, which was compared using an unpaired t test.

We compared the data obtained from NPFF cells with the values that we previously reported for GRPR cells^18^ and found several differences (Fig. 11 and Table 1). NPFF cells exhibited a greater incidence of tonic (30.8 vs. 2.6%) and transient (19.2 vs. 4.3%) action potential firing patterns than GRPR cells, with far fewer cells displaying single spike firing (7.7 vs. 40.2%). The incidence of delayed firing among NPFF cells was similar to that seen in GRPR cells (42.3 vs. 48.7%), while NPFF cells were never classified as reluctant firing, a pattern that was occasionally seen in GRPR cells (0 vs. 4.3%) (Fig. 11a). NPFF cells had a significantly lower rheobase current and action potential voltage threshold, and a greater action potential height and afterhyperpolarisation than GRPR cells (Fig. 11b–e). Together, these findings suggest that NPFF cells are more excitable than GRPR cells.

In terms of excitatory synaptic drive to NPFF cells, we found this to be greater than that seen in GRPR cells, with NPFF cells displaying a greater sEPSC and mEPSC frequency (Fig. 11f,g), as well as a greater mEPSC amplitude (Fig. 11h). The higher mEPSC frequency in NPFF cells is consistent with the significantly higher density of dendritic spines on these cells, compared to GRPR cells (Fig. 8e).

NPFF cells had a greater incidence of I_h_ (68.8 vs. 28.4%) and I_As_ (25.0 vs. 3.7%) but a reduced incidence of I_Ar_ compared to GRPR cells (62.5 vs. 95.1%: Fig. 11i). While there were no differences in I_h_ or I_As_ amplitude, the amplitude of the I_Ar_ was significantly lower in NPFF cells than GRPR cells (Fig. 11j).

## Discussion

Our main findings are that: (1) Cre-mediated recombination in the NPFF^Cre^ line can be used to target a subset of excitatory interneurons in SDH, including the majority of those with detectable levels of pro-NPFF; (2) pro-NPFF-expressing neurons are vertical cells, but differ from GRPR-expressing vertical cells in having a much higher density of dendritic spines; and (3) electrophysiological recording from a population that includes these cells reveals important differences from those reported for the GRPR cells.

### The NPFF^Cre^ mouse

NPFF expression defines a distinct population of excitatory interneurons in the dorsal horn^8,14,32^. However, little is known about the location of these cells in somatosensory circuits, and to allow targeting of NPFF cells, we generated a NPFF^Cre^ knock-in mouse line. As expected, crossing this with the Ai9 reporter resulted in tdTomato expression in CNS regions known to contain NPFF-expressing cells, specifically the NTS, and the SDH of both spinal cord and spinal trigeminal nucleus. Similarly, intraspinal injection of AAV.flex.GFP resulted in GFP expression that was largely restricted to excitatory interneurons in laminae I-II, and we found that both reporter and viral strategies captured the majority of cells that were pro-NPFF-immunoreactive.

However, there were three unexpected findings. Firstly, both approaches failed to label a significant fraction (15–25%) of pro-NPFF-immunoreactive neurons in SDH. Secondly, both labelled many dorsal horn neurons that lacked detectable pro-NPFF, and with the viral technique, these included cells that were captured in the GRPR^Flp^ line. Thirdly, in the NPFF^Cre^;Ai9 cross scattered tdTomato-positive cells were seen in many CNS regions that are not thought to contain NPFF-expressing cells.

The finding of pro-NPFF containing cells that were not labelled with Cre-dependent virus or reporter presumably results from lack of Cre expression in some of these cells, and this was supported by our findings with fluorescent in situ hybridisation, since we observed some cells with mRNA for *Npff* but not *iCre*. The presence of the fluorescent proteins in pro-NPFF-negative cells could result from: (1) ectopic expression of Cre in cells that never express NPFF, (2) transient expression of Cre in cells that only express NPFF during development, or (3) Cre expression in cells that generate *Npff* mRNA, but do not make the pro-NPFF protein at detectable levels. In situ hybridisation failed to distinguish between these possibilities, because the levels of *iCre* mRNA in this mouse line were relatively low and scattered transcripts were seen throughout the dorsal horn. This is in contrast to our finding with a GRPR^CreERT2^ line in which we saw much higher *iCre* transcript numbers with clear restriction to individual cells, and a high degree of co-localisation with the mRNA for *Grpr* itself^18^.

Since there is not thought to be significant expression of NPFF in adult forebrain, the scattered tdTomato-labelled cells seen in regions such as caudatoputamen, neocortex and hippocampus in the NPFF^Cre^;Ai9 mouse might have resulted from either ectopic or transient expression of Cre. However, in the spinal cord the tdTomato and GFP expression (seen with reporter and viral approaches, respectively) was restricted to excitatory interneurons in the SDH, an area known to contain many NPFF-expressing glutamatergic cells^8,14^. In addition, transient expression of Cre would not account for the presence of many GFP-labelled cells that lacked pro-NPFF in the NPFF^Cre^;Ai9 mice injected with AAV.flex.GFP. It is therefore likely that the fluorescent proteins were associated with genuine *Npff* mRNA expression, even though many of these cells may not show pro-NPFF-immunoreactivity. This mismatch could result from production of low levels of pro-NPFF, below our detection threshold, or from other mechanisms, such as failure to translate the mRNA, or rapid degradation of either mRNA or protein^43,44^.

### NPFF expression in vertical cells

The reconstruction of Brainbow-labelled neurons showed that, like SDH neurons with *Grpr* mRNA^18^, these were vertical cells. However, they differed from the GRPR vertical cells in having a ~ twofold higher density of dendritic spines. In the Brainbow experiments, we were able to confirm with immunohistochemistry that the reconstructed cells contained pro-NPFF. However, this approach could not be applied to the electrophysiological experiments, and for these we used the GRPR^Flp^;NPFF^Cre^ cross to exclude those cells that were also labelled through Flp-mediated recombination (i.e. also GRPR-positive), since we have shown that the vast majority of cells with *Grpr* mRNA are not pro-NPFF-immunoreactive^18^. Although this would give a broader population than those analysed in the Brainbow experiments, we found that these cells had a significantly higher mEPSC frequency than we had previously reported for GRPR cells^18^. Together, these findings suggest that the NPFF cells that lack GRPR have a considerably higher density of excitatory synaptic input than the GRPR cells. We also found that the NPFF cells had a much lower rheobase, suggesting that they would be more excitable than the GRPR cells. Despite the apparently greater excitability of NPFF cells, we had previously reported that following noxious heat, pinch or intradermal injection of pruritogens, between 88 and 95% of GRPR neurons (identified in GRPR^CreERT2^;Ai9 mice) contained phosphorylated extracellular signal-regulated kinases (ERK)^18^, whereas the same stimuli generated phospho-ERK in a lower proportion of pro-NPFF-immunoreactive cells (30–33% for noxious heat and pruritogen injection, 50% for pinch)^14^. This discrepancy could be due to differences in the sources of excitatory synaptic input to the two populations, or in the extent to which action potential firing led to phosphorylation of ERK. Häring et al. reported that Glut9 was one of 3 of their transcriptomic clusters that expressed the NPY Y1 receptor, and consistent with this we found that Y1 receptor agonist caused outward currents in all of the NPFF cells tested. We have also tested 10 GRPR cells and found that none of these responded (ACD, unpublished observations), suggesting that the Y1 receptor is preferentially expressed by the NPFF class of vertical cells. The NPFF cells may therefore contribute to the antinociceptive action of NPY acting via Y1 receptors^42^.

Vertical cells constitute a well-defined population of excitatory interneurons in the SDH. Cells of this morphological type were originally identified in the spinal trigeminal nucleus by Gobel^45^, who named them stalked cells, based on the numerous stalk-like branches and spines on their dendrites, and proposed that they provided excitatory synaptic input to lamina I projection neurons. Grudt and Perl^9^ identified a broader population that included Gobel's stalked cells, but named these vertical cells based on the predominant orientation of their dendrites. Subsequent studies have confirmed that many vertical cells have axons that enter lamina I^9,12,38,41^, and that their postsynaptic targets include ALS projection neurons in this lamina^12,46^. This excitatory synaptic input from vertical cells to lamina I ALS neurons has formed an integral part of many of the neuronal circuits that have been proposed to explain processing of pain information at the spinal level^1,47–52^. For example, it has been suggested that vertical cells can transmit information from A-low threshold mechanoreceptors to lamina I projection cells, either directly^53,54^ or through a circuit involving other excitatory interneurons^47^. It is thought that this pathway is normally held closed by feedforward inhibition, and that loss of this inhibition leads to tactile allodynia^47,53^. However, vertical cells may also transmit other types of sensory information, including input from Aδ and C afferents that likely include nociceptors^12^, to projection neurons.

Consistent with the proposed vertical cell to projection neuron circuit, Mu et al^35^ used optogenetics to demonstrate that some lamina I projection neurons were directly innervated by GRPR cells. Little is known about the targets of the NPFF cells, although their axons form a dense plexus in laminae I and IIo^14,33,34^, and we have found that some lamina I neurons that were retrogradely labelled from the parabrachial area received numerous contacts from pro-NPFF-immunoreactive axons (RQ, unpublished observations). This suggests that both of these classes of vertical cell innervate some projection neurons in lamina I.

Given the morphological and electrophysiological differences that we observed between the NPFF and GRPR cells, it is likely that these have somewhat different roles in somatosensory processing. In particular, GRPR cells are strongly implicated in itch^55–58^, although we find that they also respond to noxious stimuli and that activating them appears to cause both pain- and itch-related behaviours^18^. In future studies, it will be helpful to characterise cells that express both NPFF and GRPR, and determine whether these show features in common with either the remaining NPFF or GRPR cells. In addition, it will be important to determine whether the NPFF and GRPR cells target the same or different ALS lamina I neurons, and to explore the behavioural effects of selectively activating or silencing these two populations.

## Methods

### Animals

All experiments were approved by the Ethical Review Process Applications Panel of the University of Glasgow, and were performed in accordance with the European Community directive 86/609/EC and the UK Animals (Scientific Procedures) Act 1986. The study was carried out in compliance with the ARRIVE guidelines.

The NPFF^Cre^ knockin mouse line was generated by Taconic Biosciences GmbH (Leverkusen, Germany), using a conventional ES cell targeting strategy and homologous recombination. Briefly, the sequence for the T2A peptide and the open reading frame of improved Cre recombinase (iCre) were inserted between the last amino acid and the translation termination codon in exon 3 of the NPFF gene. A positive selection marker (Puromycin resistance) flanked by FRT sites was removed by crossing NPFF^Cre^ mice with germline Flpe mice. The presence of the T2A sequence should result in co-translational cleavage between the NPFF and iCre proteins, resulting in coexpression of both proteins, under control of the *Npff* promoter.

For some experiments, the NPFF^Cre^ mice were crossed with either the Ai9 reporter line (Jackson Laboratory; Stock number 007909), in which Cre-mediated excision of a STOP cassette results in expression of the red fluorescent protein tdTomato, or with a mouse line (GRPR^Flp^) in which the codon-optimised form of Flp recombinase (Flpo) is knocked into the *Grpr* locus^59^.

Mice of both sexes, aged 5 to 12 weeks and weighing between 15 and 25 g, were used in this study.

### Intraspinal AAV injections

Intraspinal injections of viral vectors were performed as described previously^28^. Briefly, mice were anaesthetised with isoflurane (1% to 2%) and received injections into the lumbar spinal cord. These were targeted either into the L3 and L5 segments on one side, or the L3 segment bilaterally. The L3 and L5 spinal segments were injected through the T12/T13 and T13/L1 intervertebral spaces, respectively. Spinal injections were performed by making a small slit in the dura and inserting a glass micropipette (outer/inner tip diameter: 60/40 µm) attached to a 10 µL Hamilton syringe, 400 µm lateral to the midline and 300 µm below the pial surface. The AAV constructs that were used are listed in Table S1.

A total volume of 300 nL of virus was infused per injection site (or 500 nl for AAV-Brainbow2) at a rate of 30–40 nL/minute using a syringe pump (Harvard Apparatus, MA, USA). To minimise leakage of the injected virus, pipettes were left within the spinal cord for 5 min, and the wound was then closed. All animals that underwent surgical procedures received perioperative analgesia (0.5 mg/kg buprenorphine and 5 mg/kg carprofen).

### Immunohistochemistry

Immunohistochemistry was performed on tissue from NPFF^Cre^, NPFF^Cre^;Ai9 and NPFF^Cre^;GRPR^Flp^ mice, most of which had received intraspinal injection of AAVs, as described above. The mice were deeply anaesthetised with pentobarbitone (20 mg i.p.) and perfused with fixative that contained 4% freshly depolymerised formaldehyde. Lumbar spinal cord segments were removed from all of these animals and stored in the same fixative for 2 h at 4˚C. Brains were removed from some of the NPFF^Cre^;Ai9 mice and post-fixed in the same way.

Multiple-labelling immunofluorescence reactions were performed as described previously^18^ on 60 μm thick transverse or parasagittal sections of spinal cord, and on 100 μm thick coronal sections of brain, all of which were cut with vibrating blade microtomes (Leica VT1200 or VT1000). The sources and concentrations of antibodies are listed in Table S2. Sections were incubated for 3 days at 4˚C in mixtures of primary antibodies diluted in PBS that contained 0.3 M NaCl, 0.3% Triton X-100 and 5% normal donkey serum, and then overnight in species-specific secondary antibodies (Jackson Immunoresearch, West Grove, PA, USA), which were raised in donkey and conjugated to Alexa488, Alexa647, Rhodamine Red, Pacific Blue or biotin. All secondary antibodies were diluted 1:500 in the same diluent, apart from those conjugated to Rhodamine Red or Pacific Blue, which were diluted 1:100 and 1:200, respectively. Biotinylated antibodies were revealed either with Pacific Blue conjugated to avidin (1:1,000; Life Technologies, Paisley, UK) or with a tyramide signal amplification (TSA) method (TSA kit Cyanine 5 NEL705A001; Perkin Elmer Life Sciences, Boston, MA, USA). The TSA reaction was used to detect PPTB. Following immunoreaction, sections were mounted in anti-fade medium and stored at – 20 °C. They were scanned with either a Zeiss LSM710 confocal microscope with Argon multi-line, 405 nm diode, 561 nm solid state and 633 nm HeNe lasers, or with a Zeiss LSM900 Airyscan confocal microscope with 405, 488, 561 and 640 nm diode lasers. Confocal image stacks were obtained through a 20 × dry lens (numerical aperture, NA, 0.8), or through 40 × (NA 1.3) or 63 × (NA 1.4) oil immersion lenses with the confocal aperture set to 1 Airy unit or less. All analyses were performed with Neurolucida for Confocal software (MBF Bioscience, Williston, VT, USA).

Transverse sections of brain and spinal cord from NPFF^Cre^;Ai9 mice were reacted with antibodies against mCherry and pro-NPFF. In some cases, antibody against NeuN was included to reveal neurons, while in other cases sections were counterstained with DAPI to reveal nuclei. To quantify the proportion of neurons that were labelled with tdTomato, we used a modified disector method^28^ (with a 10 μm separation between reference and look-up section) to identify NeuN-positive cells in 3 sections each from the L4 segments of 2 mice (one female, one male), while the observer was blind to the tdTomato channel. This channel was then revealed, allowing us to determine the proportion of neurons that were labelled. To quantify overlap between pro-NPFF and tdTomato and to look for possible expression of tdTomato in inhibitory neurons, we reacted additional sections from these animals with antibodies against mCherry, pro-NPFF, NeuN and Pax2. In confocal scans of this tissue, we initially viewed only the pro-NPFF and NeuN channels, and identified all pro-NPFF-immunoreactive cells in 3 sections from each mouse. We checked for the presence or absence of tdTomato in these cells, and identified any cells that were tdTomato-positive and lacked pro-NPFF. We then assessed all tdTomato cells for the presence of Pax2.

We compared the distribution of labelling for tdTomato and GFP in 4 female NPFF^Cre^;Ai9 mice that had received injections of AAV.flex.GFP targeted on the L3 and L5 segments on one side. Transverse sections from the L3-L5 segments of these animals were reacted with antibodies against GFP, mCherry and pro-NPFF. In some cases, the reactions also included the NeuN antibody, while in others DAPI was used as a nuclear counterstain. Three sections from each animal were examined and initially all pro-NPFF-immunoreactive neurons were identified. We then viewed the tdTomato and GFP channels and looked for the presence of these fluorescent proteins in pro-NPFF-immunoreactive neurons. Finally, neurons that lacked pro-NPFF and had most of the cell body located within the section were examined for the presence of tdTomato and/or GFP.

Tissue from other NPFF^Cre^;Ai9 mice that had received injections of AAV.flex.GFP into the L3 and L5 segments on one side was used to assess overlap between tdTomato or GFP and each of three neuropeptides or their precursors. Sections were reacted with antibodies against tdTomato, GFP and either neurotensin, preprotachykinin B (PPTB) or pro-cholecystokinin (pro-CCK). In each case, 3 sections from 3 different (2 female, 1 male) mice were analysed. All neurons that were tdTomato and/or GFP-positive were identified, and then examined for the presence of the neuropeptide (precursor). A similar approach was used to determine whether any of the fluorescent protein-labelled cells were inhibitory, on sections from 2 of these animals (one female, one male) that had been reacted with antibodies against tdTomato, GFP and Pax2. These were analysed in the same way.

Four NPFF^Cre^;GRPR^Flp^ mice (3 female, 1 male) that had received injections of AAV.flex.GFP and AAV.FRT.tdTom targeted on the L3 and L5 segments on one side were used to investigate the relationship between GRPR-expressing neurons and those expressing Cre in the NPFF^Cre^ line. Because the GRPR gene is located on the X chromosome, we used male mice that were hemizygous or female mice that were homozygous for the mutated *Grpr* allele. Transverse and sagittal sections from the midlumbar spinal cords of these mice were reacted with antibodies against GFP, tdTomato and pro-NPFF. Analysis was performed on 3 transverse sections from each animal. Initially, all cells labelled with GFP and/or tdTomato were identified, and these were then assessed for the presence of pro-NPFF.

Four NPFF^Cre^ mice (2 female, 2 male) that had received injections of both Brainbow viruses^36^ into the L3 and L5 segments on one side were used to investigate dendritic morphology of the NPFF cells. Parasagittal sections were reacted with antibodies against TagRFP, mTFP, mCherry and pro-NPFF. We analysed 30 Brainbow-labelled cells in the SDH (5–9 from each mouse). These were selected based on the following criteria: (1) presence of pro-NPFF in the cell body, (2) sufficiently strong labelling with at least one of the Brainbow fluorescent proteins to allow reconstruction of fine processes. They were excluded if it appeared likely that substantial parts of the dendritic tree were not present in the section being examined. These cells were scanned and analysed as described previously^13,18,60^.

### Fluorescent in situ hybridisation

Fluorescent in situ hybridisation was carried out with RNAscope probes and RNAscope Multiplex Fluorescent Assay v2 (Catalogue number 323100, ACD Biotechne, Newark, CA 94,560, USA). Fresh frozen lumbar spinal cords from 3 NPFF^Cre^ mice (1 female, two males) were embedded in OCT medium and cut into 12 μm thick transverse sections with a cryostat (Leica CM1950; Leica, Milton Keynes, UK). Sections were mounted non-sequentially (so that sections on the same slide were at least 4 apart) onto SuperFrost Plus slides (48,311–703; VWR; Lutterworth, UK) and air dried. Reactions were performed according to the manufacturer’s recommended protocol with a number of minor refinements. Slides were fixed for 1 h (rather than 15 min) in 4% PFA and RNAscope Protease IV was applied for 15 (rather than 30) minutes prior to hybridisation. The probe combination consisted of *Npff* (Catalogue number Mm-Npff 479,901) and *iCre* (Catalogue number iCre 423,321). RNAscope positive and negative multiplex controls (Catalogue numbers 320,881 and 320,871, respectively) were run on separate sections to verify the absence of non-specific signal. Hybridisation products were visualised using TSA Vivid fluorophores (Tocris, Abingdon, United Kingdom). TSA Vivid 520 was used for the Npff probe at a concentration of 1:750. TSA Vivid 570 was used for the iCre probe at a concentration of 1:20 K. Sections were mounted with Prolong‐Glass anti‐fade medium with NucBlue (Hoechst 33,342) (ThermoFisher Scientific, Paisley, UK) and scanned with the Zeiss LSM900 Airyscan confocal microscope through the 40 × oil-immersion lens.

### Electrophysiology

Electrophysiological recordings of NPFF cells were performed on spinal cord slices that were obtained from 18 NPFF^Cre^;GRPR^FlpO^ mice (7 female, 11 male), that had received intraspinal injections of a combination of AAV.Flex.GFP and AAV.Frt.mCherry, between 1 and 3 weeks previously. As described above, we used male mice that were hemizygous or female mice that were homozygous for the mutated *Grpr* allele. Mice were deeply anaesthetised with pentobarbitone (20 mg i.p.), perfused with ice-cold dissection solution and the lumbar region of the spinal cord was isolated and embedded in low melting point agar (~ 3%; ThermoFisher Scientific). Parasagittal (300 μm) or transverse (300 μm) slices were cut with a vibrating blade microtome (7000smz-2; Campden Instruments, Loughborough, UK). Immediately after cutting, slices were incubated in an *N*-methyl-D-glucamine (NMDG) based recovery solution at 32 °C for ~ 15 min and were then placed in a modified recording solution at room temperature for an additional 1 h before being transferred to the recording chamber, where they were continually perfused with recording solution at a rate of ~ 2 ml/min. The solutions used contained the following (in mM): Dissection, 3.0 KCl, 1.2 NaH_2_PO_4_, 0.5 CaCl_2_, 7.0 MgCl_2_, 26.0 NaHCO_3_, 15.0 glucose, 251.6 sucrose; NMDG recovery, 93.0 NMDG, 2.5 KCl, 1.2 NaH_2_PO_4_, 0.5 CaCl_2_, 10.0 MgSO_4_, 30.0 NaHCO_3_, 25.0 glucose, 5.0 Na-ascorbate, 2.0 thiourea, 3.0 Na-pyruvate, 20.0 HEPES; Modified recording, 92.0 NaCl, 2.5 KCl, 1.2 NaH_2_PO_4_, 2.0 CaCl_2_, 2.0 MgSO_4_, 30.0 NaHCO_3_,25 glucose, 5.0 Na-ascorbate, 2.0 thiourea, 3.0 Na-pyruvate, 20.0 HEPES; Recording, 125.8 NaCl, 3.0 KCl, 1.2 NaH_2_PO_4_, 2.4 CaCl_2_, 1.3 MgCl_2_, 26.0 NaHCO_3_,15 glucose. All solutions were bubbled with 95% O_2_ / 5% CO_2_.

Neurons were visualised using a fixed stage upright microscope (BX51; Olympus, Southend-on-Sea, UK) equipped with a 40X water immersion objective, infrared differential interference contrast (IR-DiC) illumination, and a CCD camera (QImaging Retiga Electro; Teledyne Photometrics, Birmingham, United Kingdom). Fluorescently labelled cells were visualised using a 470 nm or 550 nm LED (pE-100; CoolLED, Andover, UK). To restrict our recordings to NPFF neurons that did not express GRPR, targeted whole-cell patch-clamp recordings were made from cells in the SDH that were GFP-positive and mCherry-negative. Patch-clamp pipettes were pulled from borosilicate glass (Warner Instruments, Holliston, MA) on a Sutter P1000 puller (Sutter Instruments, Novato, CA). These had a typical resistance of 4 to 8.5 MΩ when filled with an intracellular solution containing (in mM): 130.0 K-gluconate, 10.0 KCl, 2.0 MgCl_2_, 10.0 HEPES, 0.5 EGTA, 2.0 ATP-Na, 0.5 GTP-Na, and 0.2% Neurobiotin, pH adjusted to 7.3 with 1.0 M KOH. Data were recorded and acquired with a Multiclamp 700B amplifier and pClamp 10 software (both Molecular Devices, Wokingham, UK), and were filtered at 4 kHz and digitised at 10 kHz.

After achieving stable whole-cell configuration, the presence of spontaneous action potential firing was assessed by recording for 1 min in current clamp at resting membrane potential. Cells were voltage clamped at − 70 mV, and a series of 100 ms voltage steps from − 70 to − 50 mV (2.5 mV increments) was applied to determine the current–voltage relationship, which was used to calculate the resting membrane potential. Cells that had a resting membrane potential that was more depolarised than − 30 mV were excluded from all analysis. A series of five − 5 mV steps (1 s duration), from a holding potential of − 70 mV, was used to calculate input resistance.

To characterise action potential firing patterns, a series of 1 s depolarising current steps of increasing amplitude (5 pA increments) were delivered in current clamp mode, in some cases a bias current was applied to hold the membrane potential at around − 60 mV. Firing patterns were defined on the basis of previously published criteria^9,13,41,61–64^ as follows: tonic firing if they exhibited a continuous action potential discharge throughout the step; transient if the action potential discharge occurred only during the early part of the step; delayed if there was a clear delay between the start of the depolarising step and the first action potential, and single spike if only 1 or 2 action potentials occurred at the onset of the step. The properties of action potentials for each neuron were measured from the first action potential that occurred at rheobase.

Subthreshold voltage-activated currents were investigated by applying a voltage step protocol, where cells were held at − 60 mV followed by a 1 s − 90 mV step and then a 200 ms − 40 mV step. Automated leak subtraction was applied to remove capacitive and leak currents. This protocol enables the identification of 2 types of transient outward current and 2 types of inward currents. The outward currents that are associated with the − 90 to − 40 mV depolarising step are consistent with A-type potassium currents (I_A_) and are classified as rapid (I_Ar_) or slow (I_As_) on the basis of their kinetics. A transient inward current seen during this depolarising step is considered to reflect the low-threshold “T-type” calcium current (I_Ca,T_). A slow inward current during the − 60 to − 90 mV hyperpolarising voltage step is classified as the hyperpolarisation-activated current (I_h_). The I_A_ amplitude was measured as the peak amplitude of the transient outward current. The amplitude of the I_h_ was measured during the final 200 ms of the hyperpolarising step, and inward currents that were greater than − 5 mV were classified as I_h_.

Excitatory synaptic drive to NPFF cells was assessed by voltage-clamping cells at − 70 mV and recording sEPSCs and mEPSCs, the latter in the presence of tetrodotoxin (TTX) (0.5 µM), bicuculline (10 µM) and strychnine (5 µM). To determine whether NPFF cells received excitatory synaptic input from TRPV1-expressing primary afferents, mEPSCs were recorded prior to and during application of the TRPV1 agonist, capsaicin (2 µM). EPSC data were analysed using Mini Analysis (Synaptosoft). The events were detected automatically by the software and were then rejected or accepted after visual examination. Neurons were considered to receive input from capsaicin-sensitive (TRPV1-expressing) afferents if capsaicin application resulted in a significant leftward shift in the distribution of interevent intervals, indicating an increase in frequency, or nonresponsive if this threshold was not reached.

The response of NPFF cells to the neuropeptide Y1 receptor agonist, [Leu^32^,Pro^34^]-NPY (300 nM), was investigated by voltage-clamping cells at − 50 mV and bath applying the agonist, in the presence of TTX, bicuculine and strychnine. Recordings were made for a total of 8 min, which comprised a 1 min baseline period that was followed by a 4 min application of [Leu^32^,Pro^34^]-NPY and then a further 3 min in the absence of the agonist. Cells were considered to be responsive to [Leu^32^,Pro^34^]-NPY if the slow outward current during any 30 s section of the recording was at least 5 pA greater than the mean current recorded during the 1 min baseline period. The input resistance of cells was monitored throughout the recording by applying a − 5 mV step (20 ms duration) every 30 s. The effect of [Leu^32^,Pro^34^]-NPY on input resistance was determined by comparing the input resistance calculated during the 1 min baseline with the final minute of [Leu^32^,Pro^34^]-NPY application.

All chemicals were obtained from Sigma, except sucrose, glucose, NaH_2_PO_4_ (Thermo Fisher Scientific), NaCl, KCl, HEPES (VWR, Lutterworth, United Kingdom), TTX (Alomone, Jerusalem, Israel), bicuculline, [Leu^32^,Pro^34^]-NPY (Tocris, Abingdon, United Kingdom), and Neurobiotin (Vector Laboratories, Peterborough, United Kingdom).

### Statistical analysis

Recorded neurons were classified as responsive to capsaicin by comparing the cumulative probability distribution of mEPSC interevent intervals with a 2-sample Kolmogorov–Smirnov test. Changes in EPSC frequency between sEPSCs and mEPCSs, recorded in the same cell, and between the input resistance of cells recorded at baseline and during [Leu^31^,Pro^34^]-NPY application were compared using Wilcoxon signed rank tests. Electrophysiological properties of NPFF cells were compared with those previously recorded in GRPR cells^18^ by using Mann–Whitney U tests, except after-hyperpolarisation, which was compared using an unpaired t-test. The differences in spine density between NPFF and GRPR cells were compared using an unpaired t-test. Data are expressed as mean ± SD, and *P* values of less than 0.05 were considered significant. Statistical tests were performed in Prism version 9 (GraphPad Software, San Diego, CA).

## Supplementary Information

Supplementary Information.

## Data Availability

Data can be accessed from an open repository at the following link: 10.5525/gla.researchdata.1418.
