# Supplementary material for: Characterisation of NPFF-expressing neurons in the superficial dorsal horn of the mouse spinal cord
Source: Sci Rep. 2023 Apr 11;13:5891. doi: 10.1038/s41598-023-32720-3 (PMC10090074; doi:10.1038/s41598-023-32720-3)
Supplement: Supplementary file 1 — Supplementary Information. [file 41598_2023_32720_MOESM1_ESM.pdf]

Characterisation of NPFF-expressing neurons in the superficial dorsal horn of the mouse spinal cord

Raphaëlle Quillet[1*,3], Allen C. Dickie[1,3], Erika Polgár[1], Maria Gutierrez-Mecinas[1], Andrew M. Bell[1], Luca Goffin[1], Masahiko Watanabe[2] and Andrew J. Todd[1*]

[1]School of Psychology and Neuroscience, Sir James Black Building, University of Glasgow, Glasgow, G12 8QQ, UK; [2]Department of Anatomy, Hokkaido University School of Medicine, Sapporo 060-8638, Japan

[3]These authors contributed equally: Raphaëlle Quillet and Allen Dickie

Number of words in abstract: 198

main text: 4513

online methods: 3075

Number of references: 64

Number of figures: 11 (plus 3 supplementary)

*Correspondence:

Raphaelle Quillet, Spinal Cord Group, Sir James Black Building, University of Glasgow, Glasgow, G12 8QQ, UK. Email: raphaelle.quillet@glasgow.ac.uk

or

Andrew Todd, Spinal Cord Group, Sir James Black Building, University of Glasgow, Glasgow, G12 8QQ, UK. Email: andrew.todd@glasgow.ac.uk

Fig S1

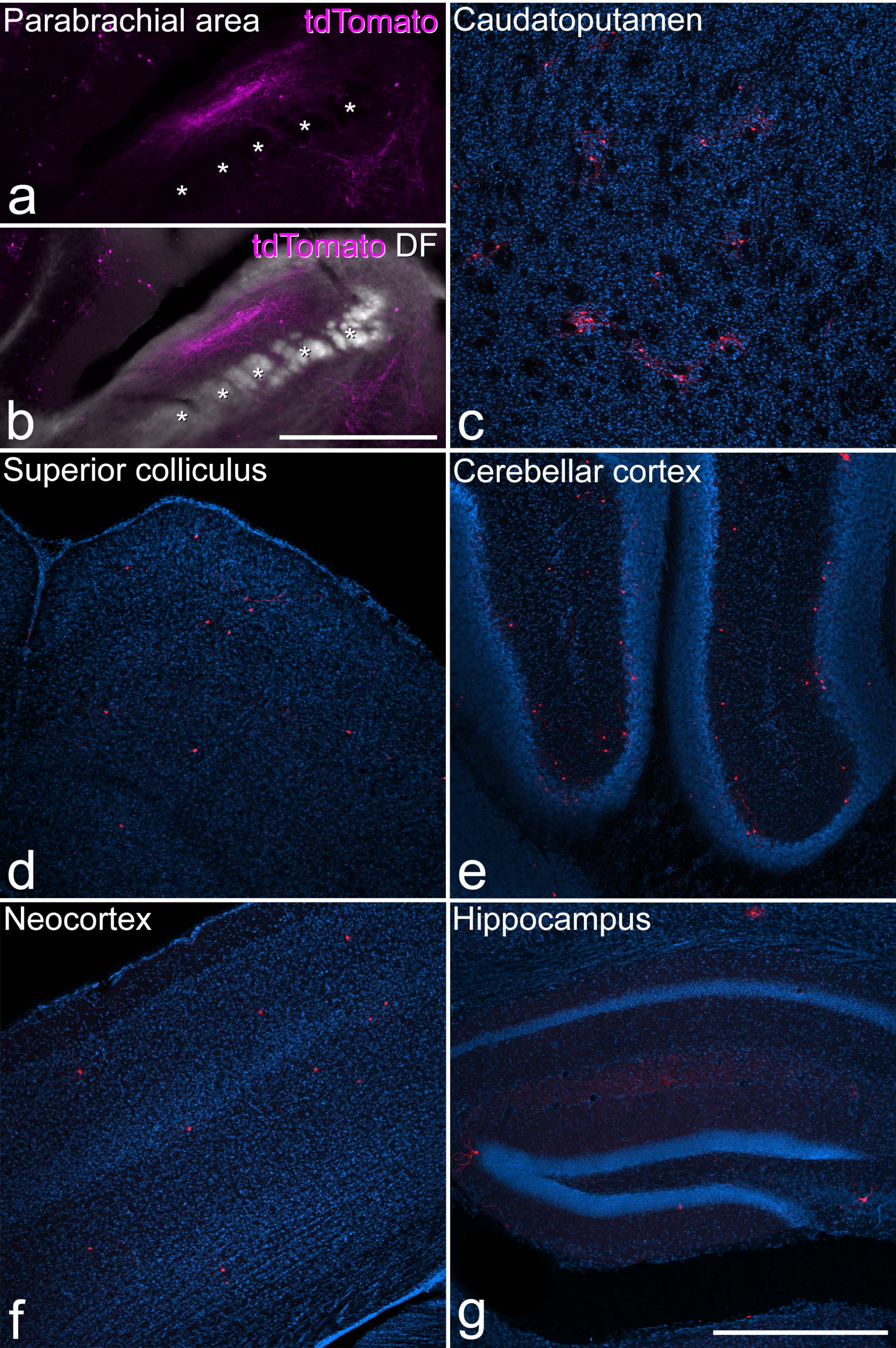

Parabrachial area
tdTomato
a
tdTomato DF
b
Caudatoputamen
c
Superior colliculus
d
Cerebellar cortex
e
Neocortex
f
Hippocampus
g

Fig S2

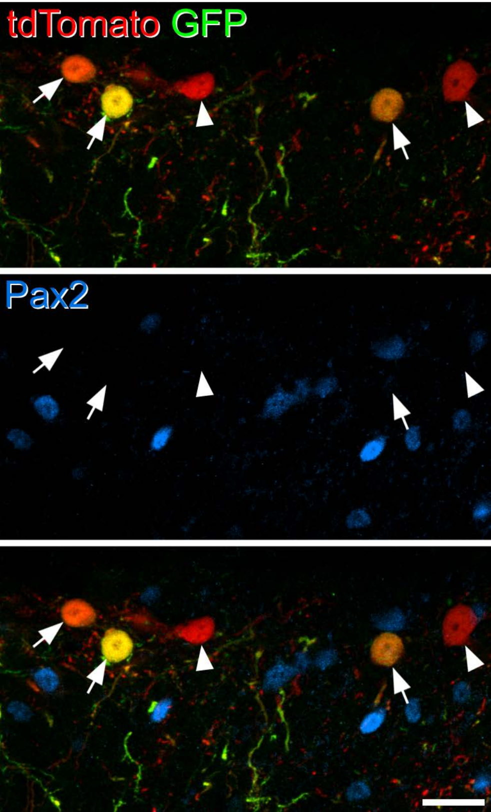

Fig S3

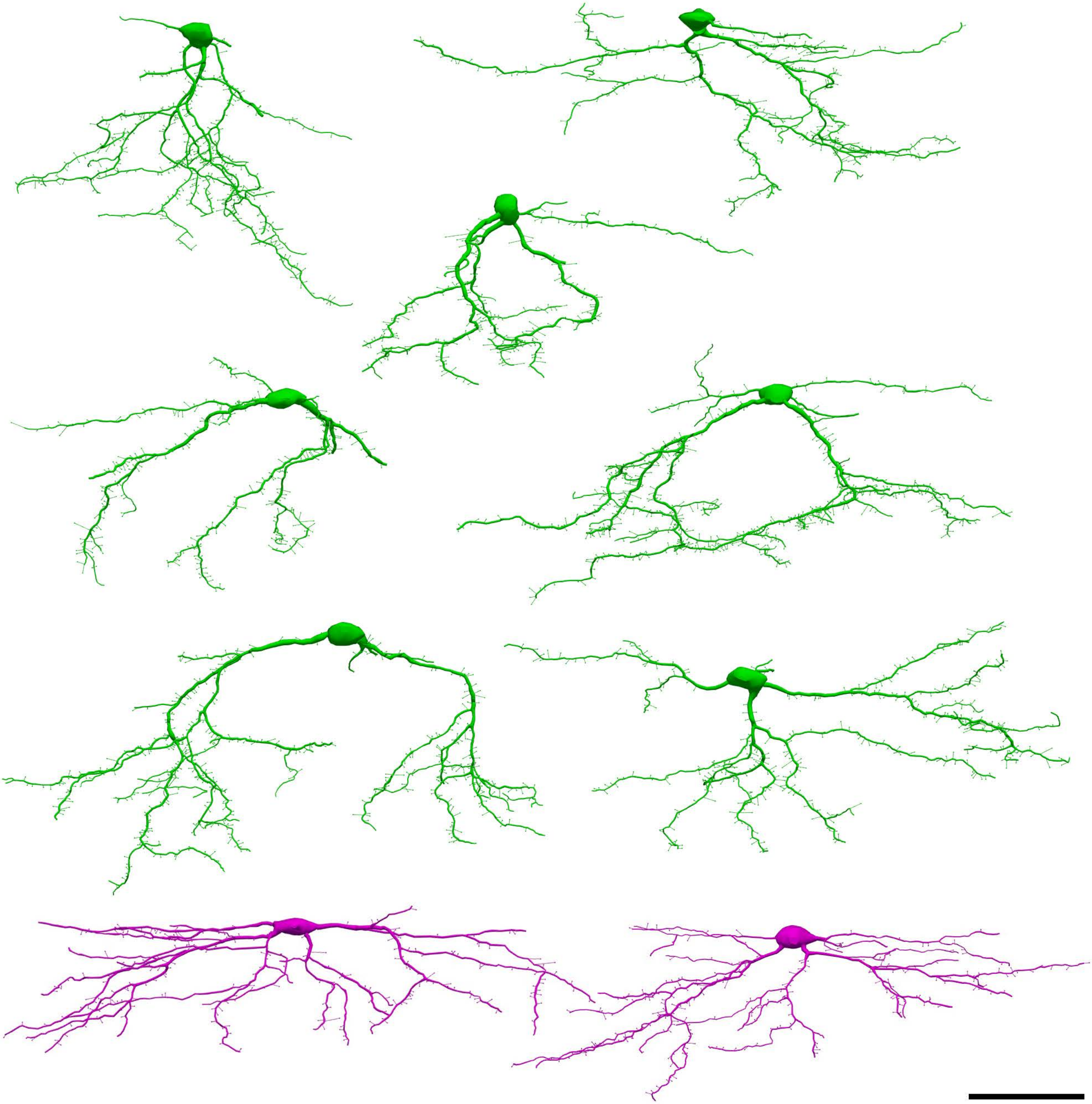

Table S1 Viruses used in this study

						Details of injection	
	**Construct(s)**	**Serotype**	**Promoter**	**Catalogue number**	**Source**	**Number of GCs**	**Volume**
**AAV.flex.eGFP**	eGFP	AAV8	CAG	v158-8	VVF Zurich	2.58×10^8	300 nl
**AAV-EF1a-BbTagBY (AAV-BB1)**	TagBFP, YFP	AAV9	hEF1a	45185	Addgene	7.55×10^7 – 3.02×10^8	500 nl
**AAV-EF1a- BbChT (AAV-BB2)**	mTFP, mCherry	AAV9	hEF1a	45186	Addgene	7.44×10^7 – 2.98×10^8	500 nl
**AAV.FRT.mCherry**	mCherry	AAV2	hSyn1	V188-2	VVF Zurich	8.7×10^8	300 nl

Table S2 Antibodies used in this study

GFP	Chicken	Abcam	ab13970	1:1000	RRID:AB_300798
mCherry*	rat	Invitrogen	M11217	1:1000	RRID:AB_2536611
PPTB	Guinea pig	Kaneko et al (1998)		1:50,000	RRID:AB_2783015
pro-NPFF	Guinea pig	M Watanabe		0.83 μg/ml	RRID:AB_2783015
Pro-CCK	Rabbit	M Watanabe		1:1000	RRID:AB_2571674
Neurotensin	Rabbit	Immunostar	20072	1:5000	RRID:AB_572254
Pax2	Rabbit	Sigma	HPA047704	1:200	RRID:AB_2636861
mTFP	Rat	Kerafast	EMU103	1:500	
TagRFP**	Rabbit	Ximbio	155266	1:500	
NeuN	Mouse	Merck	MAB377	1:500	RRID:AB_2298772

*The mCherry antibody also recognises tdTomato

** The antibody against tagRFP also recognises tagBFP

Supplementary figure legends

Fig S1 Expression of tdTom in other brain areas in $NPFF^{Cre}$;Ai9 mouse. **a**,**b** show axonal labelling for tdTomato (magenta) in the parabrachial area. The location of the superior cerebellar peduncle (marked with asterisks) can be seen in the dark field (DF) image in **b**. **c**-**g**: scattered tdTomato-positive cells (magenta) are present in several brain areas, including the caudatoputamen, superior colliculus, cerebellar cortex, neocortex and hippocampus. In all cases the sections were counterstained with DAPI (blue) to reveal cell nuclei. **a**, **b** are from a single confocal optical section, while **c** to **g** are from 5, 7, 7, 9, and 20 optical sections at 4 µm z-separation. Scale bars: **a**, **b** = 500 µm, **c**-**g** = 500 µm.

Fig S2 Lack of Pax2 staining in $NPFF^{Cre}$ cells. Pax2-immunostaining (blue) in a section from a $NPFF^{Cre}$;Ai9 mouse that had been injected with AAV.flex.GFP. Five cells that are tdTomato positive (red) are present in this field. Three of these (arrows) are also positive for GFP (green), and therefore appear orange or yellow in the merged image, while the other 2 (arrowheads) lack GFP. None of these cells are Pax2-immunoreactive. Images are maximum intensity projections of 3 confocal optical sections at 1 µm z-spacing. Scale bar = 20 µm.

Fig S3 The upper part of the figure shows 7 of the Brainbow-labelled NPFF-immunoreactive cells (green) that were reconstructed with Neurolucida for this study. For comparison, we also include 2 GRPR cells (magenta) that were examined as part of a previous study (Polgár et al, *Pain* **164**, 149-170, 2023). Note that the

positions of all dendritic spines are shown, but the sizes of spine heads and shapes of spine necks do not represent the actual sizes and shapes of these structures.
